# Climate-driven spatiotemporal dynamics of *Aedes* infestation and dengue transmission in Porto Alegre, Southern Brazil

**DOI:** 10.1371/journal.pntd.0014201

**Published:** 2026-08-24

**Authors:** Adryan Aparecido da Silva, Álvaro Gil Araujo Ferreira, José Lourenço, Amanda Cupertino de Freitas

**Affiliations:** 1 Faculty of Pharmacy, Universidade Federal de Minas Gerais, Belo Horizonte, Minas Gerais, Brazil; 2 Mosquitos Vetores, Instituto René Rachou – Fiocruz Minas, Belo Horizonte, Minas Gerais, Brazil; 3 Universidade Católica Portuguesa, Católica Medical School, Católica Biomedical Research Centre, Oeiras, Portugal; 4 Ecovec, Belo Horizonte, Minas Gerais, Brazil; Sichuan University, CHINA

## Abstract

Dengue transmission is strongly influenced by climatic conditions that affect mosquito population dynamics and virus circulation. In Southern Brazil, where dengue historically occurred at low levels, recent climatic anomalies may be contributing to the expansion of *Aedes* vectors and increased local dengue incidence. This study investigated the spatiotemporal association between climatic variables, *Aedes* infestation and dengue cases in Porto Alegre (Southern Brazil, from 2018 to 2025). Entomological, surveillance and climatic data were analyzed using Moran’s I and LISA for spatial association, Kendall correlation, polynomial regression and LASSO to identify relevant drivers and develop predictive models of mosquito infestation and dengue incidence. Strong spatial association between *Aedes aegypti* and *Aedes albopictus* was observed, with persistent local clusters detected across all years. Annual climatic variables were associated with mosquito abundance in several districts. Overall, rainfall frequency had a stronger effect on *Ae. aegypti* abundance than accumulated rainfall. Temperature and lagged infestation indices showed strong associations with both species and dengue incidence, with lag effects detected up to eight weeks. Predictive models demonstrated good agreement between observed and predicted values, particularly at low to moderate infestation levels. Lagged variables were consistently retained in both mosquito abundance and dengue incidence models, highlighting the importance of temporal predictors in anticipating vector dynamics and dengue risk. This approach is broadly applicable for predicting *Aedes* infestation and disease incidence and it underscores the importance of integrating entomological and climatic surveillance data to enhance anticipation and early detection of dengue risk periods in large urban settings and inform more effective public health interventions.

## Author summary

Dengue is a viral disease transmitted by mosquitoes that affects millions of people worldwide. Its spread is strongly influenced by climatic conditions that modulate mosquito life cycles. In Southern Brazil, where dengue has historically occurred at low levels, recent climate variability and extreme weather events may be creating favorable conditions for the expansion of *Aedes* mosquitoes and increased local dengue transmission. In this study, we analyzed mosquito, dengue, and climatic surveillance data in Porto Alegre, Brazil, from 2018 to 2025 to understand how climate influences mosquito infestation and dengue incidence. We found that mosquito infestation and dengue cases show clear spatial patterns across the city and are strongly influenced by temperature and rainfall. Interestingly, the number of rainy days had a stronger effect on mosquito abundance than the total amount of rainfall. Higher mosquito infestation was associated with increased dengue cases in the following eight weeks. These findings show that combining climatic and mosquito surveillance data can help anticipate periods of higher dengue risk and support more effective mosquito control and public health actions.

## Introduction

Dengue, one of the most important mosquito-borne diseases globally, is well documented in tropical and subtropical regions, causing outbreaks in South America, Southeast Asia and the Western Pacific [1,2]. However, recent events associated with climate change, globalization and urbanization have shifted its geographic distribution towards higher latitudes [3,4].

Mosquito reproduction, longevity and vector competence for viral transmission depend on climatic conditions [5–8]. *Ae. aegypti* and *Ae. albopictus,* the key vectors of dengue, have a short life cycle, with full development from egg to adult within a few of weeks [9]. Consequently, their populations can be affected both by long-term seasonal weather patterns (e.g., temperature, humidity) or short-term extreme events such as floods, heavy rainfall, and heat waves [10,11].

*Ae. aegypti* was historically restricted to the African continent, whereas *Ae. albopictus* was restricted to Southeast Asia. Both species have undergone major geographic expansion over the past century, largely driven by globalization processes (e.g., increased human mobility) and environmental changes that create new suitable habitats [12,13]. Currently, *Ae. aegypti* has a near-global distribution and is considered the primary dengue vector in urbanized regions [14–16]. *Ae. albopictus*, in turn, is recognized for its greater ecological plasticity and tolerance to a wider range of climatic conditions, and was first reported in Brazil in 1986 in Rio de Janeiro and Minas Gerais (Southeast region) [17,18].

In 2024, Brazil reported nearly 6.5 million suspected dengue cases, representing almost half of the cases reported in the Americas [19,20]. The virus is widely distributed across the country, with all four serotypes identified, indicating hyperendemicity [21,22]. The current epidemiological scenario of dengue in Brazil presents a unique challenge, as predicting local transmission depends on the complex interplay among human, climatic and mosquito factors [23–25].

In the first three months of 2022, 63 municipalities detected local dengue transmission for the first time, with half of them in Southern Brazil. This region has historically been associated with low *Aedes* infestation levels and dengue incidence but has recently experienced both increased mosquito presence and dengue activity. Recent evidence suggests that environmental changes may be creating suitable conditions for both mosquitoes and viruses, potentially facilitating dengue emergence in this region of the country [22–24].

Despite growing evidence linking climatic conditions to mosquito population dynamics and dengue transmission, important knowledge gaps remain, particularly in temperate regions where dengue has historically been limited. Understanding how climatic variability influences vector infestation and disease dynamics at the local scale is essential for improving surveillance and early warning systems.

Porto Alegre, the capital city of Rio Grande do Sul, Brazil’s southernmost state, has experienced extreme weather events in recent years, including temperature anomalies in 2023, flooding in 2024 and heavy rainfall in 2025. This study investigates the relationship between climatic variability, mosquito infestation and dengue incidence in Porto Alegre between 2018 and 2025 through an integrated analysis of entomological, epidemiological and climatic surveillance data.

## Methods

### Study region

Porto Alegre is one of Brazil’s metropolitan areas, located in Southern Brazil, with 1,388,794 inhabitants (2022). It covers an area of 495.39 km², with nearly 50% urbanization. The climate is temperate, with well-defined seasons.

### Entomological data

Data were obtained from MosquiTRAPs as part of the municipal surveillance system in Porto Alegre. Trap were installed in strategic locations based on local dengue cases and mosquito infestation (S1 Fig). Numbers of adult female *Aedes* adults were assessed weekly and RT-PCR was performed to detect dengue virus (DENV) serotypes (DENV1–4) in pooled samples. All infestation data were normalized by the number of traps installed in the same period. The DENV-positive Trap Index was calculated as the number of traps with dengue virus detection divided by the total number of traps installed per year × 100. The Mean Female *Aedes* Index (MFAI) was calculated as the number of adult female *Aedes* mosquitoes captured per week divided by the total number of traps inspected in the same week.

### Climatic data

Climatic data were sourced from the Copernicus Data Store [26], a repository maintained by the European Centre for Medium Range Weather Forecasts (ECMWF) with daily resolution for the Etc/GMT-3 time zone at the location of Porto Alegre. Air temperature at 2 m above ground level and dew point were converted to degrees Celsius by subtracting 273.15. Weakly and annual aggregated values were calculated from daily observations and subsequently averaged. Relative humidity was calculated using the following formula:


$$\begin{array}{c} Relative\;humidity=\text{1}\text{0}\text{0}\times \frac{e^{\frac{\text{1}\text{7}.\text{6}\text{2}\text{5}\cdot T_{dew}}{\text{2}\text{4}\text{3}.\text{0}\text{4}+T_{dew}}}}{e^{\frac{\text{1}\text{7}.\text{6}\text{2}\text{5}\cdot T}{\text{2}\text{4}\text{3}.\text{0}\text{4}+T}}} \end{array}$$

Precipitation was converted to millimeters and aggregated to obtain cumulative values per week and year. For the analysis of accumulated days with precipitation, 1mm of rainfall was used as the threshold to define a rainy day.

### Viral-mosquito climatic suitability

To evaluate climatic conditions that promote transmission, the climate-driven mosquito-viral suitability Index P was obtained from a publicly available global synthetic dataset [27]. To assess the joint effect of mosquito-viral suitability and vector infestation, the Vector-Fitness Infestation Index was defined as:


$$\begin{array}{c} VFII\;=\;MFAI\;x\;Index\;P \end{array}$$

### Land cover data

Land cover data for the study area data were obtained from MapBiomas Brasil [28]. Each raster was downloaded and reprojected to match the coordinate reference system of the Porto Alegre municipal boundary shapefile. The rasters were then cropped and masked to the study area to retain only pixels within the municipality limits. The resulting rasters were converted into tables containing pixel coordinates and land cover class codes for each year. Land cover classes were grouped into two broader categories: anthropic land uses (urbanized areas, pasture, agriculture, rice, sugarcane, land uses) and vegetation cover (forest formation, savanna formation, grassland formation, and planted forest), according to the MapBiomas classification scheme.

Because anthropic land use and vegetation cover showed minimal variation during the study period, these variables were not included in the statistical analyses (S2a–S2b Fig).

### Dengue cases

Dengue is a notifiable disease in Brazil and cases are reported to the Notifiable Diseases Information System (SINAN) of the Brazilian Ministry of Health. Cases are initially notified based on clinical evaluation of symptoms, while a subset is confirmed by laboratory diagnosis.

Incidence data for suspected dengue cases between 2018 and 2025 were obtained from InfoDengue [29], an open Brazilian surveillance platform that integrates data from SINAN. Information on all autochthonous cases during the same period was retrieved from the official Power BI epidemiological dashboard maintained by the municipality of Porto Alegre [30].

### Statistical analysis

#### Spatial autocorrelation.

For each year, spatial analyses of *Ae. aegypti*, *Ae. albopictus*, DENV+ Traps Index and dengue cases were performed using Moran’s Global Index (Moran’s I) [31] and Local Indicators of Spatial Association (LISA) to assess geographic association and clustering in the study area. A neighborhood matrix was constructed using k-nearest neighbors (KNN) with the four nearest neighbors identified for each unit based on Euclidean distances between centroid coordinates.

Positive values of Moran’s I indicate spatial clustering, values close to zero suggest a random spatial pattern and negative values indicate spatial dispersion. Statistical significance was determined using a permutation test.

LISA identifies local clusters by classifying each spatial unit as High-High or Low-Low, indicating areas surrounded by similar values (clusters), or High-Low or Low-High, indicating areas with values that contrast with those of neighboring areas (spatial outliers). While Moran’s I provides a global measure of spatial correlation, LISA identifies the specific locations driving spatial dependence.

### Correlation and modeling

#### Kendall correlation.

Associations between climatic variables, entomological indicators and dengue incidence were evaluated using Kendall’s rank correlation coefficient (τ). To explore local variability in climate-entomology relationships across district within year, Kendall correlation was calculated separately for each district. To increase temporal resolution, weekly correlations were computed. Variables lagged from zero to eight weeks were also included to account for potential delayed effects.

#### Polynomial regression.

To assess the effect of rainfall frequency on mosquito infestation, 1 mm of rainfall was defined as a rainy event and 5, 10 and 30-day time windows were used to represent short-, medium- and long-term rainfall persistence, respectively. For each epidemiological week, MFAI was compared with rainfall frequency over the preceding period. First to fourth order polynomial regression models were then fitted to evaluate non-linear relationships between variables. The optimal model was selected based on the Akaike Information Criterion (AIC), with lower values indicating better model fit while accounting for model complexity. Additionally, analysis of variance (ANOVA) was conducted to assess whether increasing polynomial degree significantly improved model performance. For the 5-day model degree 2 produced the lowest AIC (2895.248) and significantly improved model fit compared with degree 1 (ANOVA F = 7.66, p = 0.00567), whereas higher-order terms were not significant (degree 2 vs. 3: F = 0.50, p = 0.478; degree 3 vs. 4: F = 1.21, p = 0.270). Therefore, a second-degree polynomial was selected. For the 10-day model, degree 2 substantially reduced the AIC (2859.943 vs. 2870.921 for degree 1) and significantly improved fit (ANOVA F = 12.99, p = 0.0003). The addition of a third-degree term did not significantly improve the model (degree 2 vs. 3: F = 0.067, p = 0.794). Consequently, a second-degree polynomial was retained. For the 30-day model, progressive reductions in AIC were observed with increasing polynomial degree (degree 1: 2746.532; degree 2: 2670.353; degree 3: 2666.402; degree 4: 2635.155). ANOVA comparisons also indicated significant improvements up to 4 (degree 1 vs. 2: F = 79.26, p < 0.001; degree 2 vs. 3: F = 5.949, p = 0.0148; degree 3 vs. 4: F = 33.39, p < 0.001). Therefore, a fourth-degree polynomial was selected.

#### LASSO.

To model associations between climatic variables, entomological indicators and dengue incidence, Least Absolute Shrinkage and Selection Operator (LASSO) regression was employed. This method applies a penalty that shrinks less informative variables to zero, thereby reducing model complexity and mitigating multicollinearity. Three LASSO models were fitted: two models to address weekly mosquito infestation based on lagged climatic variables and a third to evaluate the joint effect of Index P, MFAI and VFII for both species on weekly log (dengue incidence + 1).

The lambda value that minimized the cross-validated error was used to penalize the coefficients. A Gaussian distribution was assumed and a rolling validation framework was applied to train the model. An initial training window of 50 weeks was adopted because it encompasses approximately one complete annual cycle, allowing the model to capture baseline seasonal dynamics while preserving a sufficiently long prospective evaluation period. Model performance was evaluated using the root mean squared error (RMSE), compared to a linear model, and the deviance ratio relative to the intercept-only model.

All analyses were conducted using R (version 4.4.1). Spatial analysis was performed using the R package *spdep.* LASSO analysis, Kendall and polynomial regression were performed with the *glmnet* and *stats* R packages.

## Results

### Porto Alegre spatio-temporal *Aedes* surveillance from 2018 to 2025

**Fig 1:**
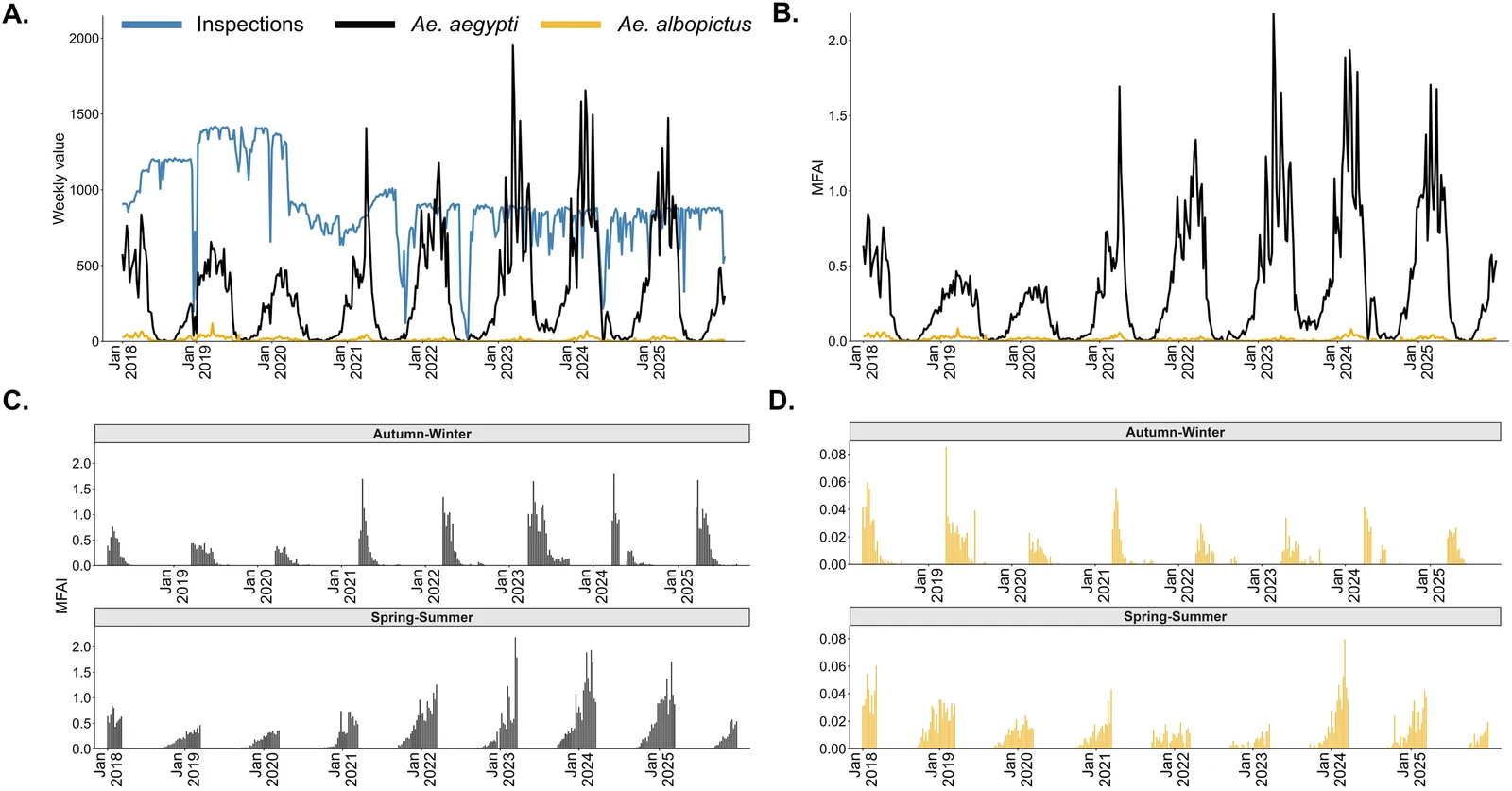
Temporal dynamics of mosquito surveillance indicators in Porto Alegre, Brazil, from 2018 to 2025. **(a)** Weekly values for inspections, *Ae. aegypti* and *Ae. albopictus* in Porto Alegre. **(b)** Weekly Mean Female *Aedes* Index (MFAI) for both species. **(c)** MFAI of *Ae. aegypti* in autumn-winter (upper panel) and spring-summer (lower panel). **(d)** MFAI of *Ae. albopictus* in autumn-winter (upper panel) and spring-summer (lower panel).

Across the period, approximately 50 districts were under surveillance (S3a Fig). 1,447 distinct traps were installed, capturing 130,122 females of *Ae. aegypti* and 4,618 females of *Ae. albopictus* over 375,776 inspections. *Aedes* was the dominant genus among all mosquitoes collected (S4 Fig). Weekly inspections increased in 2019, reaching an average of 1,400 inspections per week, then decreased during 2020 and remained stable until 2025 (Fig 1a). MFAI data indicated an increase in infestation since 2021 (Fig 1b). Both absolute counts and MFAI values were significantly higher for *Ae. aegypti*, demonstrating lower capture abundance of *Ae. albopictus in* Porto Alegre.

For *Ae. aegypti,* the highest number of captured females was observed during the summer–autumn transition (Fig 1c), peaking at 1,951 mosquitoes in epidemiological week 11 of 2023. Epidemiological weeks 31 of 2021 and 2022, weeks 32 and 33 of 2022, and week 21 of 2024 were the only weeks with no captures, all occurring during the autumn–winter period.

In contrast*,* three of the four high capture weeks for *Ae. albopictus* occurred in autumn-winter (Fig 1d): epidemiological week 13, 2019 with 119 females captured, followed by weeks 16 and 17, 2018 with 62 females captured. The fourth highest peak occurred in epidemiological week 10 of 2024 (69 females).

The number of DENV-positive traps increased steadily after 2021, reaching 122 in 2025 (S3a Fig). To account for sampling effort, we calculated the annual positivity rate as the proportion of DENV-positive traps among traps with mosquito capture. This rate remained low but increased from 0.01% in 2018 and 0.02% in 2019 to 0.46% in 2022, 0.33% in 2023, and 0.40% in 2024, peaking at 0.98% in 2025. Despite the low detection rates, this trend indicates increasing viral circulation over time. Pools of trapped mosquitoes were tested for serotype identification, revealing co-circulation of DENV-1 and DENV-2 in 2023 and 2024 (S3b Fig).

The spatial analysis of infestation was conducted to detect spatial autocorrelation and clusters of *Ae. aegypti* and *Ae. albopictus* using the ratio of total female captures by the total number of traps installed per year. Spatial autocorrelation peaked in 2023 for both species (S1 Table), with *Ae. aegypti* showing strong correlation across years.

**Fig 2:**
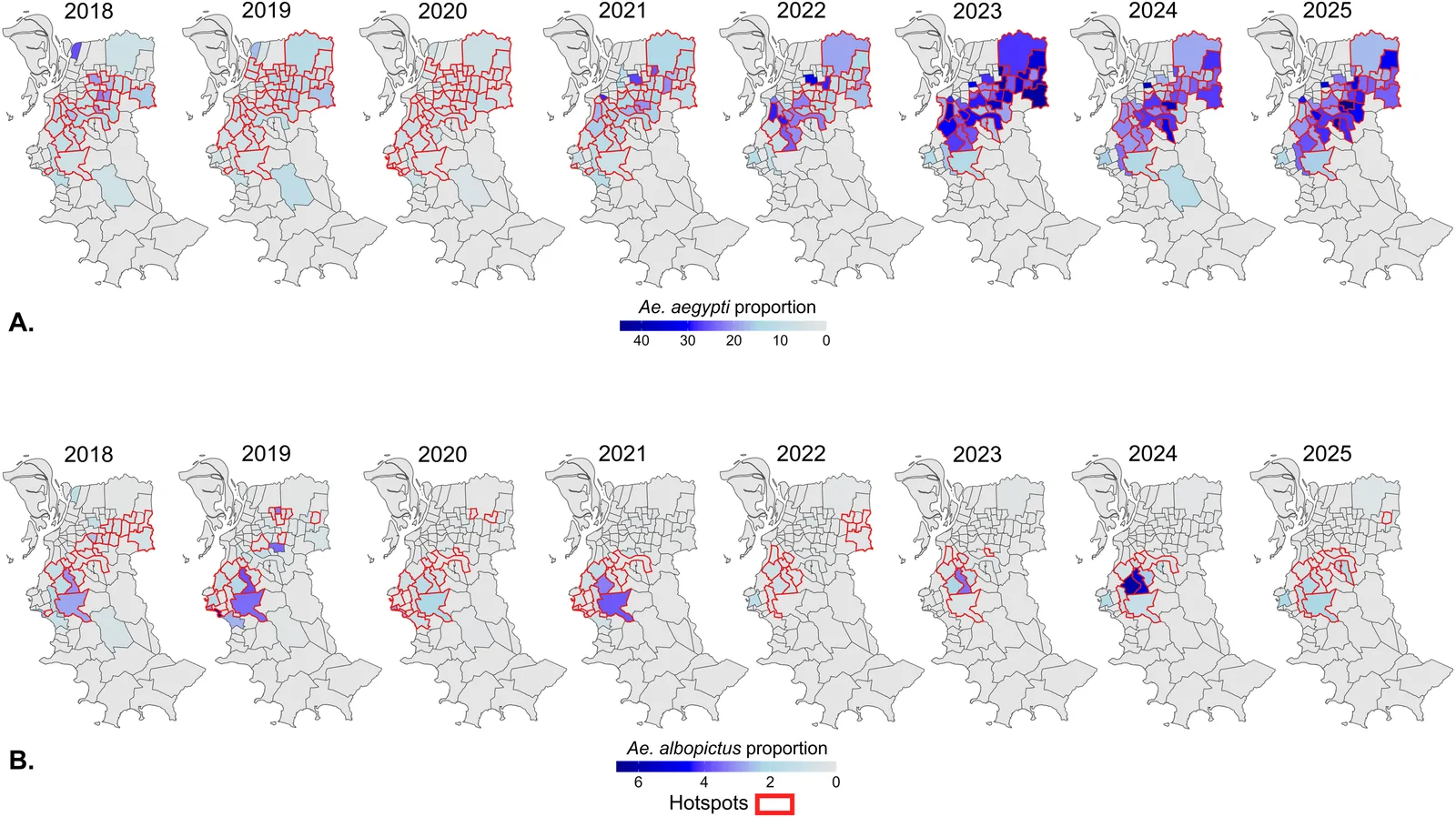
Spatiotemporal clustering of *Ae. aegypti* and *Ae. albopictus* proportions in Porto Alegre, Brazil, from 2018 to 2025. **(a)** Annual proportion of *Ae. aegypti* captured in each neighborhood from 2018 to 2025 (left to right). Proportions were calculated as the total number of mosquitoes captured divided by the total number of traps installed in each neighborhood per year. Red outlines indicate significant High–High LISA clusters (hotspots), representing neighborhoods with high values surrounded by neighboring areas with similarly high values. **(b)** Annual proportion of *Ae. albopictus* captured in each neighborhood from 2018 to 2025 (left to right). Proportions were calculated as the total number of mosquitoes captured divided by the total number of traps installed in each neighborhood per year. Red outlines indicate significant High–High LISA clusters (hotspots). Base layer: Municipality of Porto Alegre, SMAMUS Digital Maps (Neighborhood Shapefile): https://prefeitura.poa.br/carta-de-servicos/mapas-digitais-da-smamus.

In 2018 *Ae. aegypti* was concentrated in the Partenon, Northwest, Central-South, Northeast and Cruzeiro regions, subsequently expanding to additional areas through 2025. Notable clustering was observed in the North, East and Eixo Baltazar regions in recent years, with densities exceeding 40 mosquitoes per trap in these clusters (Fig 2a). Despite the presence of clusters in other areas across all years, *Ae. albopictus* remained concentrated primarily in the South and Central-South regions, with densities reaching up to 6 mosquitoes per trap in 2019 and 2024 (Fig 2b). This spatial restriction is consistent with the higher capture rates observed for *Ae. aegypti* in Porto Alegre.

### Climatic factors influence mosquito dynamics in Porto Alegre

**Fig 3:**
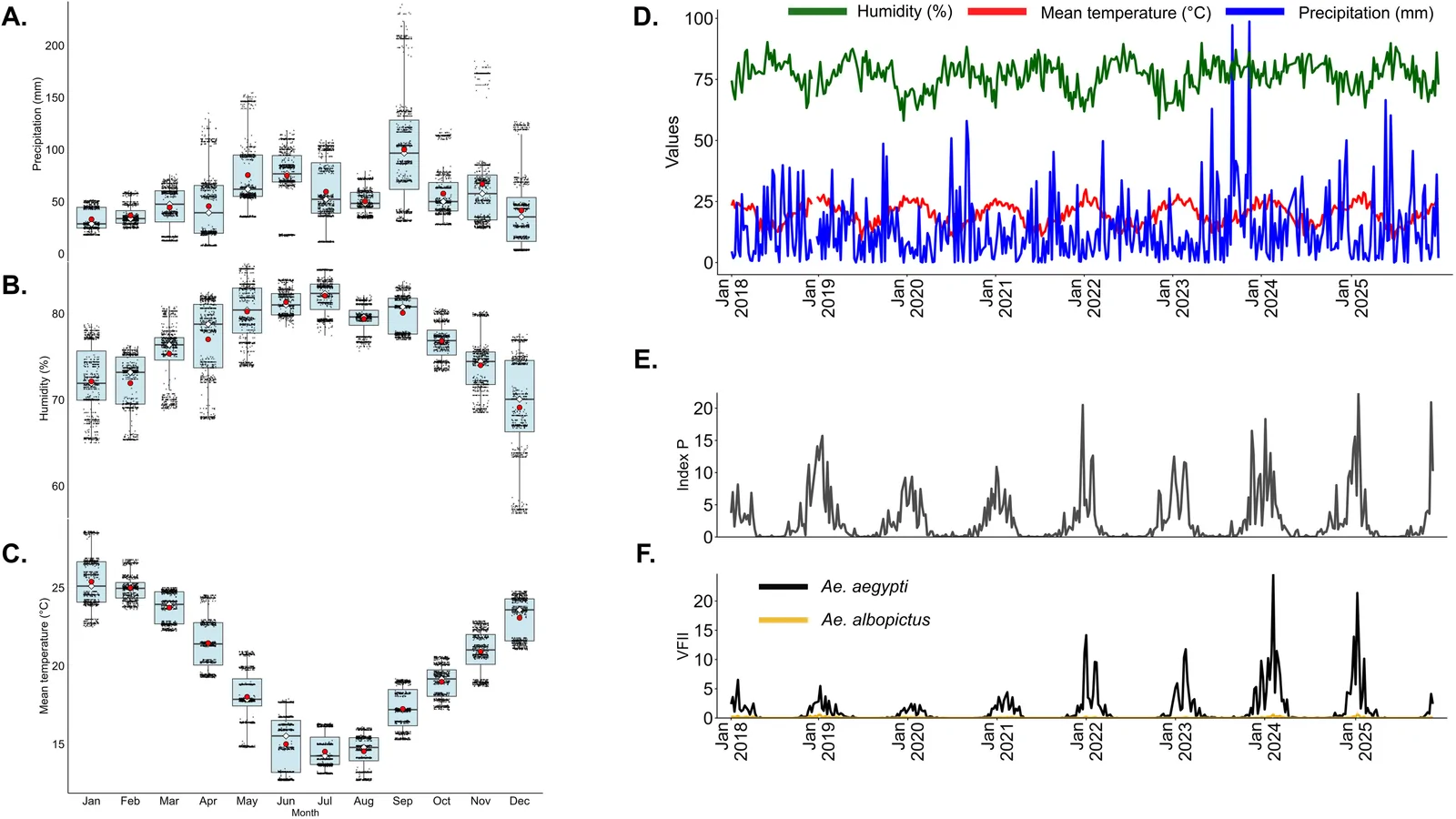
Climatic variability and vector suitability indices associated with mosquito dynamics in Porto Alegre, Brazil, from 2018 to 2025. Monthly distribution of **(a)** precipitation (mm), **(b)** relative humidity (%), and **(c)** mean temperature (°C) across districts. Each point represents the mean value per district across all years; red circles indicate the mean and white diamonds the median values. **(d)** Weekly time series of relative humidity, mean temperature, and precipitation. **(e)** Weekly Index P derived from temperature and humidity. **(f)** Weekly Vector-Fitness Infestation Index (VFII) for both species.

Porto Alegre has a temperate climate, with well-defined seasons, weather and climate conditions. Each climate variable exhibited different effects across districts. Accumulated precipitation showed spatial variability between April and June, with rainfall ranging from 10 to 100 mm, between September and December, rainfall reached 200 mm in some regions (Fig 3a).

Relative humidity showed the greatest spatial variability in December, ranging from 50% to 75%, followed by January through May (Fig 3b). Mean temperature exhibited lower spatial variability overall, with January, April, May, and June showing the highest temperature variance within the study area (Fig 3c). During winter months (June to August) temperatures fell below 15°C, a critical biological threshold for mosquito life cycles [8].

In the epidemiological weeks 36 and 46 (September and November) of 2023, rainfall reached approximately 100 mm. The highest mean temperatures occurred in January 2022 and February 2025, both reaching nearly 30°C. The highest humidity levels were observed between May and July of across all years, approaching 90% (Fig 3d).

Index P values indicated climatic suitability for dengue transmission. Suitable conditions for female *Aedes* mosquitoes in the study area occurred primarily between October and May (late spring to early autumn). Since 2023, seasonal Index P values have increased compared to previous years (Fig 3e).

To assess the combined effect of mosquito infestation and climate-driven transmission potential, the MFAI and Index P values were integrated into the Vector Fitness Infestation Index (VFII). The multiplicative formulation was chosen to represent a biological synergy, assuming that transmission risk is expected to increase substantially only when both vector abundance (MFAI) and climatic suitability (Index P) are simultaneously high, both metrics were not standardized prior to multiplication.

VFII showed the greatest transmission potential from September to April each year. The highest values were reported in epidemiological week 6 of 2025 (February), where VFII reached 24 for *Ae. aegypti* and 0.59 for *Ae. albopictus*, followed by week 3 of 2025 (January) with values of 21 and 0.61, respectively (Fig 3f). Although the Index *P* did not discriminate between species, the lower infestation of *Ae. albopictus* resulted in much lower VFII compared to *Ae. aegypti*. The previously reported increases in Index *P* over time became more apparent through VFII. A linear regression analysis revealed a significant positive temporal trend in the VFII for *Ae. aegypti* (β = 0.0038, p = 0.001), indicating a gradual increase in transmission potential due to climatic suitability over the study period. In contrast, no significant temporal trend was observed for *Ae. albopictus* (β = −0.00007, p = 0.058).

Kendall correlation analysis revealed multiple associations between background variables and mosquito species. Strong associations between climatic variables and *Aede*s infestation were observed across districts over the study period (S2 Table). Boa Vista, Jardim Itu, Jardim Leopoldina, Jardim Sabará, Vila Jardim, Três Figueiras and Mário Quintana showed strong associations between accumulated precipitation and *Ae. aegypti* infestation*.* Três Figueiras also showed a correlation with relative humidity. In Partenon and Sarandi, relative humidity and accumulated precipitation, respectively, were associated with *Ae. albopictus* infestation. In Teresópolis and Glória, increases in both relative humidity and mean temperature were associated with increased in *Ae. albopictus* infestation.

To verify the effect of lagged climatic variables on mosquito dynamics, weekly values of accumulated precipitation, mean temperature, and relative humidity were correlated with MFAI of both species. Mean temperature was the only variable positively correlated with MFAI, showing a gradual increase over time and peaked at 6 weeks. Humidity had a negative effect on the infestation index, and weekly accumulated precipitation seems to minimal influence mosquito presence (Table 1). For temperature and humidity the strongest gain in association occurred within the first four weeks, after which the increase became more gradual and approached a plateau.

**Table 1:** Kendall correlation of weekly lagged climate variables and Mean Female *Aedes* Index (MFAI). *** p < 0.001.

Weeks lag	τ					
	MFAI *- Aedes aegypti*			MFAI *- Aedes albopictus*		
	Precipitation	Temperature	Humidity	Precipitation	Temperature	Humidity
0	-0.093***	0.419***	-0.2158**	-0.1066***	0.4048***	-0.1991***
1	-0.1245***	0.4789***	-0.2339**	-0.1106***	0.4661***	-0.1986***
2	-0.1033***	0.5049***	-0.2518**	-0.1259***	0.4857***	-0.2180***
3	-0.0993***	0.5483***	-0.2681**	-0.0734***	0.5056***	-0.2323***
4	-0.094***	0.5809***	-0.2930**	-0.0852***	0.5352***	-0.2547***
5	-0.0626	0.6128***	-0.3015***	-0.0559	0.5662***	-0.2607***
6	-0.0646	0.6202***	-0.3125***	-0.0571	0.5695***	-0.215***
7	-0.0732*	0.6182***	-0.322***	-0.0567	0.5419***	-0.248***
8	-0.0729*	0.586***	-0.3173***	-0.0598	0.5101***	-0.2487***

**Fig 4:**
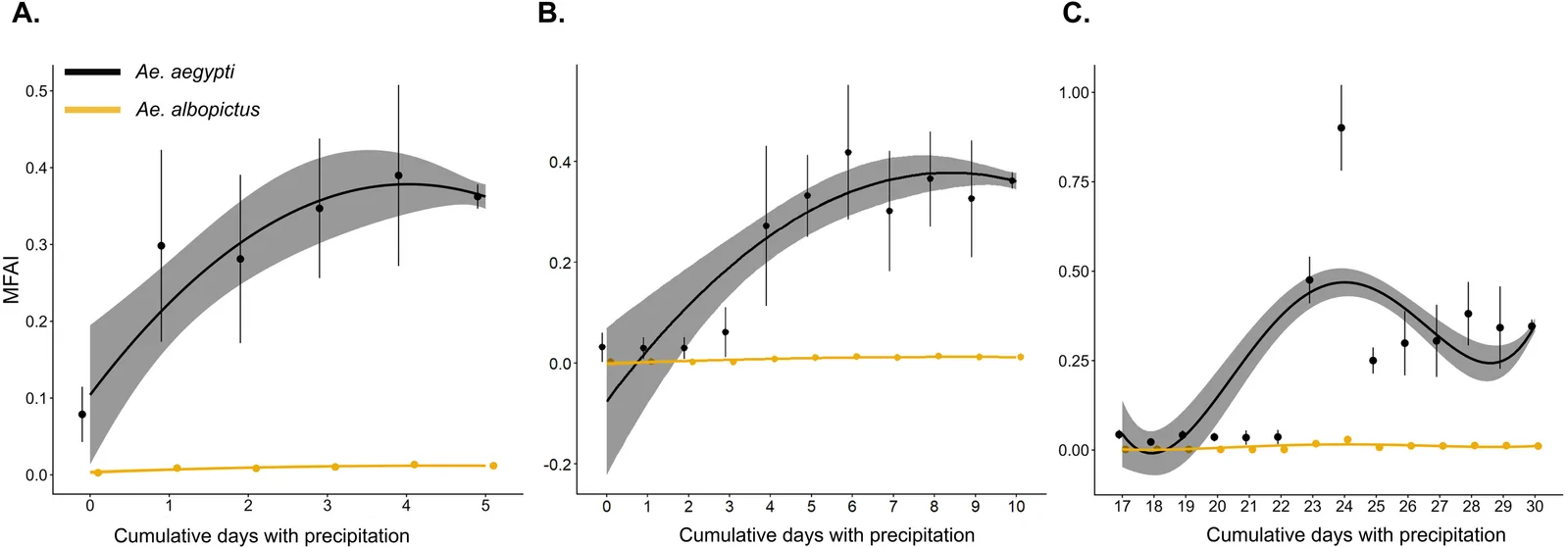
Nonlinear relationship between cumulative rainy days and Mean Female *Aedes* Index (MFAI) for *Aedes aegypti* and *Aedes albopictus.* **(a)** Association between MFAI and the cumulative number of rainy days over a 5-day period, **(b)** 10-day period, and **(c)** 30-day period. Points represent mean values and error bars indicate standard errors. Solid lines represent polynomial regression fits, with shaded areas indicating 95% confidence intervals. Second-order polynomials provided the best fit for the 5- and 10-day periods, whereas a fourth-order polynomial provided the best fit for the 30-day period.

Model selection based on AIC and ANOVA indicated that second-order polynomial models provided the best fit for the 5- and 10-day periods, whereas a fourth-order polynomial model was selected for the 30-day period. The relationship between the number of rainy days and *Ae. aegypti* MFAI showed a positive association, peaking at approximately four rainy days within a 5-day period (Fig 4a) and eight rainy days within a 10-day period (Fig 4b). Over a 30-day period, the relationship was non-linear, with MFAI increasing up to approximately 23–24 rainy days before showing a slight decline (Fig 4c). *Ae. albopictus* did not exhibit a significant association with the number of rainy days in any of the evaluated time windows. The low infestation levels of this species in the study area likely reduced the ability to detect consistent environmental relationships.

### Mosquito infestation modulates dengue dynamics in Porto Alegre from 2018 to 2025

From 2022 to 2025, the dengue incidence per 100,000 inhabitants has consistently increased (S5 Fig). Notably, the epidemiological weeks 13–21 of 2025 (March to May) reached the highest incidence levels across the period.

Autochthonous dengue cases were first detected in 2010 in Porto Alegre in Jardim Carvalho (East region). Since then, the disease has spread across the city, with diagnoses made based on clinical symptoms, and a percentage of these confirmed through laboratory analysis. Between 2019 and 2025, 72,965 dengue cases were reported, 51% of which were confirmed and 93% of these were classified as autochthonous. In 2025, Passo das Pedras district (Eixo Baltazar region) reported the highest burden of autochthonous cases, with 2,364, followed by Rubem Berta district (Eixo Baltazar region) with 2,236 cases. A weak spatial autocorrelation was detected across the study area (S3 Table). In 2024, Restinga (Restinga region) and Partenon (Partenon region) reported 1,171 and 1,029 autochthonous cases, respectively. Partenon also reported a high number of cases, with 1,377 registered across 2023, while in 2022, Jardim Carvalho registered 1,039 cases (S6a-S6d Fig).

Between 2022 and 2025, some districts exhibited localized hotspots where up to 50% of inspected traps tested positive for DENV (S6e–S6h Fig). However, overall positivity remained low when considering the total number of inspections, indicating that viral circulation was spatially heterogeneous and concentrated in specific areas rather than widespread across the city. Spatial autocorrelation of DENV-positive traps increased over time, with moderate clustering observed in 2023 that peaked in 2025 (S4 Table), coinciding with districts reporting the highest number of autochthonous dengue cases. Additionally, Kendall correlation analysis supported a positive association between *Ae. aegypti* infestation (MFAI) and autochthonous dengue cases (S5 Table).

To assess the predictive potential of MFAI for dengue incidence, Kendall correlation analysis using lagged weekly MFAI values was applied. The coefficients increased progressively with increasing lag for both species, indicating that higher mosquito infestation levels preceded increases in dengue incidence. However, most of the increase in correlation occurred within the first four weeks. For *Ae. aegypti*, **τ** increased from 0.2737 at lag 0 to 0.4953 at lag 4 (+0.2216), whereas only a further increase of +0.0991 was observed between lags 4 and 8. Similarly, for *Ae. albopictus*, **τ** increased from 0.1206 to 0.4479 during the first four weeks (+0.3273), with only a modest additional increase (+0.0802) thereafter, indicating a progressively stronger temporal association and suggesting that mosquito infestation precedes dengue occurrence (Table 2).

**Table 2:** Kendall correlation - Lagged Mean Female *Aedes* Index (MFAI) and autochthonous dengue cases. *** p < 0.001.

Weeks lag	τ	
	MFAI *- Aedes aegypti*	MFAI *- Aedes albopictus*
0	0.2737***	0.1206***
1	0.3376***	0.322***
2	0.3911***	0.3645***
3	0.4470***	0.4085***
4	0.4953***	0.4479***
5	0.5392***	0.4886***
6	0.5732***	0.5318***
7	0.5913***	0.5388***
8	0.5944***	0.5281***

### Modeling entomological infestation and dengue incidence

Predictive models were applied to evaluate the contribution of climatic and entomological variables to mosquito infestation and dengue incidence. For *Ae. aegypti* infestation, a linear model (LM) produced a cross-validated RMSE of 0.2866, whereas a LASSO model achieved 0.313, with a LASSO deviance ratio of 0.524. For *Ae. albopictus*, RMSE values were 0.01 for LM and 0.00975 for the LASSO model, with a deviance ratio of 0.493. Although improvements in predictive error were minimal, penalization reduced coefficient magnitudes among correlated climatic variables, improving model parsimony. Temperature at a three and four-week lags received the largest standardized penalized coefficient for *Ae. aegypti* and *Ae. albopictus*, respectively, indicating the strong contribution of temperature to model prediction among retained climatic variables (S6 Table).

For dengue incidence, the LM model produced an RMSE of 1.003, whereas the LASSO model produced an RMSE of 1.13. The deviance ratio was 0.616, indicating that 61% of null deviance was explained by the penalized model. MFAI of both species and VFII of *Ae. albopictus* had the highest coefficients, reinforcing the strong predictive relevance of mosquito infestation index (MFAI) (S6 Table).

**Fig 5:**
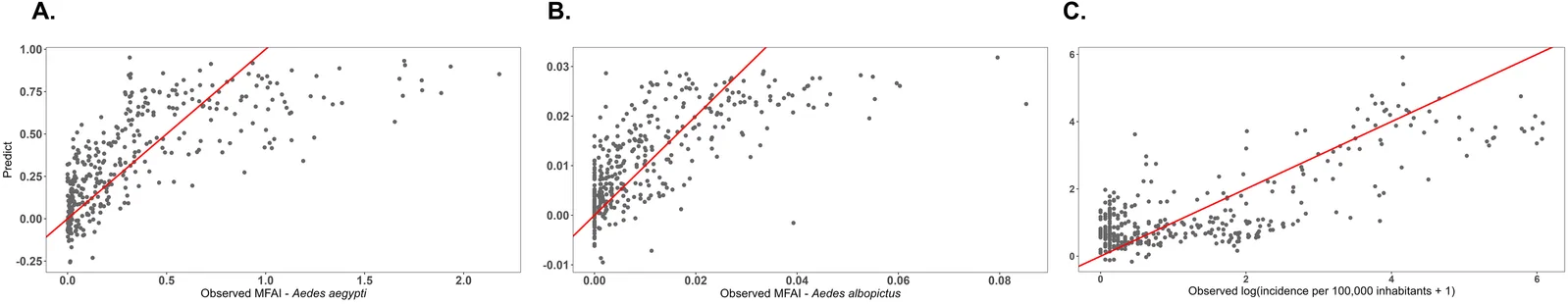
Predictive performance of LASSO regression models for mosquito abundance and dengue incidence. LASSO plots for observed versus predicted values of **(a)** MFAI – *Aedes aegypti* (RMSE = 0.313, deviance ratio = 0.524), **(b)** MFAI – *Aedes albopictus* (RMSE = 0.01, deviance ratio = 0.493) and **(c)** log(dengue incidence +1) (RMSE = 1.13, deviance ratio = 0.616. Red lines represent identity (y = x).

Compared to the unpenalized linear model, LASSO produced smaller coefficient magnitudes, indicating shrinkage of potentially inflated estimates due to multicollinearity. This improved model stability and generalizability. The *Ae. aegypti* MFAI model showed good predictive performance (Fig 5a), with an MAE of 0.19, a R² of 0.46, and a deviance ratio of 0.52. Calibration parameters were close to the ideal values, with an intercept of 0.018 (95% CI: −0.026 to 0.062) and a slope of 1.355 (95% CI: 1.224–1.486). In contrast, predictive accuracy for *Ae. albopictus* MFAI was more limited (Fig 5b), with a lower R² (0.32), a deviance ratio of 0.49, and a calibration slope of 0.807 (95% CI: 0.702–0.913), indicating reduced ability to reproduce fluctuations in infestation intensity despite low absolute prediction errors (MAE = 0.008). The dengue incidence model (Fig 5c) obtained an MAE of 0.79, a R² of 0.46, and a deviance ratio of 0.62. Calibration analysis indicated a positive intercept (0.314; 95% CI: 0.196–0.433) and a slope greater than one (1.418; 95% CI: 1.319–1.516), suggesting systematic underestimation of high-incidence periods.

## Discussion

This study examines how climatic variables drive *Aedes* infestation and influence dengue cases in Porto Alegre, Brazil. Results confirm persistent spatial clustering of *Ae. aegypti* from 2018 to 2021, as previously reported [32], with expansion into new districts between 2022 and 2025 in the Northwest, North, Cruzeiro and Central-South regions. In 2023, the year with the strongest spatial autocorrelation (S1 Table), several areas registered approximately 40 mosquitoes per trap, corresponding to the highest weekly infestation index observed in the study. Notably, this coincided with the most pronounced expansion of dengue into Southern Brazil.

Glória, South, Central-South, Cristal and Cruzeiro emerged as critical areas for *Ae. albopictus* infestation, with persistent clusters across all years. In the Central-South, districts such as Nonoai and Teresópolis, characterized by lower population density, likely provide microhabitats and peridomestic breeding sites that favor the ecological profile of this species [33,34]. In 2025, increased infestation was also observed in the Partenon region, particularly in Vila São José and Vila João Pessoa, areas marked by high population density, informal settlements, and substantial peridomestic garbage accumulation [35]. These socioenvironmental conditions may create peri-urban-like niches in densely populated settings, highlighting the ecological plasticity of *Ae. albopictus* and supporting its characterization as a predominantly peri-urban vector capable of adapting to urban environments with suitable breeding opportunities.

Annual analyses reinforced the influence of climatic variables at the district level, with three of Porto Alegre’s most populated districts (Mario Quintana, Partenon and Sarandi), showing strong correlations of accumulated precipitation and relative humidity to both *Aedes* species, while temperature was correlated with *Ae. albopictus* in Teresópolis and Glória.

Regarding the influence of weekly climatic variables on mosquitoes, temperature showed an increasingly strong association with *Aedes* abundance over time. The mosquito traits relevant to dengue transmission peak between 23 and 34°C [36] and temperatures below 18°C are expected during winter in most of Southern Brazil. However, high MFAI was also observed in autumn, indicating sustained vector reproduction at lower temperatures, and suggesting adaptation that support disease transmission year-round [37,38]. The association peak obtained at 6 weeks is compatible with previous evidence indicating that in ~74% of the territory in Brazil, dengue incidence tends to peak at four week lag of ecological suitability (25% of the territory) or at eight week lag (49% of the territory) [39].

Weekly relative humidity showed a weak negative correlation with population growth, while accumulated precipitation showed negligible effects. In contrast, the number of rainy days revealed a positive association with *Ae. aegypti* at 5- and 10- day window. Similar results have been reported in another temperate city [40], where consecutive rainy days likely maintain stability of breeding sites and boost aquatic development, while disruptive rainfall may flush larvae and eggs from containers, reduce adult flight activity, and impair the dispersion of oviposition attractants, thereby decreasing trap captures [41,42]. Taken together, these results indicate that annual climatic variables reflect broader environmental suitability, whereas weekly weather fluctuation may alter short-term infestation dynamics.

Dengue local transmission relies on a complex interplay among climatic variables, herd-immunity, human movements and exposure, and vector and DENV presence. An increase in mosquito density does not immediately translate into dengue cases, as the virus must complete its extrinsic and intrinsic incubation period before symptom onset. Additionally, delays in accessing the healthcare system and in case reporting likely explain the progressive correlation observed between mosquito infestation and autochthonous dengue cases [43]. *Ae. aegypti* and *Ae. albopictus* are also responsible for transmission of Zika virus (ZIKV) and Chikungunya virus (CHIKV) in Porto Alegre [43]. Nevertheless, CHIKV and ZIKV incidence were minimal, with sporadic cases reported (S7a, S7b Fig). This can be explained by insufficient introduction of those viruses to spark outbreaks, or by the lower infestation of *Ae. albopictus*, which determines their lower transmission potential [44].

Since 2022, VFII has increased seasonally for *Ae. aegypti,* indicating mosquito-viral capacity for disease transmission in these periods. Peaks were observed in 2024, when Brazil registered more than 6 million dengue cases [45], the highest number on record, and in 2025, the year with the highest incidence level in Porto Alegre. Further analysis should include the joint effect of mosquito infestation and viral transmission suitability on reported dengue incidence in Porto Alegre and elsewhere.

The application of a LASSO predictive model allowed the integration of multiple variables and assessment of the joint effect on mosquito infestation and dengue incidence, reducing collinearity and shrinking variables to zero. Previous work reported its performance considering only precipitation variables on *Aedes* abundance [46]. In this study, LASSO was extended to consider weekly lagged mean temperature, accumulated precipitation, and relative humidity effects on MFAI. Residual prediction across all models showed a consistent pattern: low observed values were frequently overestimated, whereas high values were consistently underestimated.

Empirical 95% prediction intervals derived from the out-of-sample residual distribution are presented in S8. For the dengue model (S8a Fig), observed incidence values were largely encompassed by the prediction intervals, indicating that the model adequately captured the overall temporal dynamics of dengue transmission. Interestingly, relatively wide prediction intervals were also observed during the pre-endemic period (January 2019– December 2021, weeks 51–206), despite the lower incidence values. This pattern results from the use of intervals based on the global residual distribution obtained throughout the entire rolling validation period. Consequently, the uncertainty bands incorporate prediction errors observed during subsequent large incidence periods and therefore represent conservative estimates of uncertainty during low-transmission periods. This behavior reflects the highly heterogeneous and episodic nature of dengue transmission.

For the *Ae. aegypti* MFAI model (S8b Fig), prediction intervals remained relatively narrow and closely tracked the observed temporal fluctuations, supporting the good predictive performance and calibration metrics obtained for this model. In contrast, the *Ae. albopictus* MFAI model (S8c Fig) showed broader uncertainty to reproduce changes in infestation intensity, consistent with its lower predictive R² and calibration metrics. Nevertheless, most observations remained within the prediction intervals, indicating that the estimated uncertainty adequately encompassed the observed variability. Although, direct comparisons between mosquitoes models should be interpreted with caution, as *Ae. albopictus* has considerably lower infestation levels than *Ae. aegypt*i, resulting in reduced variability in the entomological data and thus lower predictive performance. Additionally, extreme infestation and incidence levels (i.e., peaks and troughs) may depend on variables not included in the present modeling framework. Consequently, while the selected lagged climatic and entomological predictors capture the dominant temporal structure of transmission dynamics, incorporating additional factors may be necessary to accurately reproduce extreme-magnitude events.

Interestingly, the MFAI of *Ae. albopictus* presented a strong negative penalized coefficient (S6 Table), whereas its VFII showed a positive coefficient in the dengue incidence model, although with smaller magnitude. This may reinforce the previous result that *Ae. albopictus* infestation alone is more prevalent in peri-urban contexts with lower dengue transmission, while its ecological fitness under favorable climatic conditions enhances its epidemiological relevance. These findings underscore the importance of distinguishing between mosquito presence and the potential for climate-driven viral transmission when modeling transmission dynamics, suggesting that climatic suitability enhances the epidemiological relevance of *Ae. albopictus* in the study area.

Separate models were developed to demonstrate whether VFII provides additional predictive value. The results showed that the MFAI of *Ae. aegypti* consistently outperformed all other models (RMSE = 1.17, MAE = 0.82, R² = 0.42, deviance ratio = 0.51). In contrast, models based on Index P alone (predictive R² = −0.07; deviance ratio = 0.04) and VFII (predictive R² = −0.02 and −0.09; deviance ratios = 0.10 and 0.03 for *Ae. aegypti* and *Ae. albopictus,* respectively) showed substantially poorer predictive performance. Therefore, under the epidemiological conditions of Porto Alegre, mosquito abundance represented by MFAI was the primary driver of predictive performance, and the addition of Index P did not provide incremental predictive value.

Predictive models combining weekly climate and entomological indicators may serve as an early-warning component for disease surveillance systems and provide a powerful framework for public health planning for outbreak mitigation. Such integration can support targeted mosquito control interventions, optimize resource allocation across districts, and enhance preparedness during years of heightened climatic suitability such as periods with unusually warm temperatures or persistent rainfall.

Nevertheless, some limitations impact the generalization of the study. External validation in independent geographic settings was not performed. In addition, dengue surveillance data obtained from the InfoDengue system may be affected by underreporting, notification delays, and temporal variations in case ascertainment. Finally, while dengue incidence was modeled as cases per 100,000 inhabitants, thereby accounting for population size, the analyses did not include population distribution, demographic characteristics, or human mobility variables, which may contribute to spatial heterogeneity in dengue transmission.

The main contribution of this study does not lie in methodological innovation, but rather in its application to an emerging public health challenge in Brazil. Dengue has expanded rapidly into the southern states, where a large proportion of the population remains susceptible to infection. Despite this recent epidemiological shift, relatively few studies have investigated the environmental and entomological drivers of dengue emergence in these regions, particularly in large urban centers and temperate areas such as Porto Alegre.

These findings highlight the importance of integrating climatic information with routine vector surveillance to improve the understanding and anticipation of dengue transmission dynamics in temperate urban settings. As dengue continues to expand into regions historically considered at low risk, such as southern Brazil, integrated surveillance systems will play a key role in the early detection of transmission risk and in supporting timely public health responses under ongoing climatic and environmental change.

## Supporting information

S1 Fig Spatial location of MosquiTRAPs in Porto Alegre from 2018 to 2025. Data were obtained from the latitude and longitude values of each distinct trap. Base layer: https://prefeitura.poa.br/sites/default/files/usu_img/planejamento_urbano/Mapas%20Digitais/Shapefile_Bairros.zip
(PNG)

S2 Fig Land cover of Porto Alegre, Southern Brazil. All the data and classes were obtained from Mapbiomas Brasil. Urbanized area, Pasture, Agriculture, Rice, Sugarcane, Mining and Other classes are anthropic land use. Forest formation, Savana formation, Grassland formation and Planted forest classes are vegetation land use. Base layer: https://prefeitura.poa.br/sites/default/files/usu_img/planejamento_urbano/Mapas%20Digitais/Shapefile_Bairros.zip
(PNG)

S3 Fig MosquiTrap dengue virus surveillance in Porto Alegre. (a) Gray line stands for districts under surveillance and red line for dengue positive traps per year from 2018 to 2025. (b) dengue serotypes obtained from positive traps per year from 2018 to 2025; orange bars are referent to DENV-1 and pink ones to DENV2.
(PNG)

S4 Fig Mosquito genus captured per year from 2018 to 2025. Proportion of captured mosquitoes was calculated as the ratio of total captured female of both mosquito genus (*Aedes* and *Culex*) among all captures.
(PNG)

S5 Fig Weekly dengue incidence per 100,000 inhabitants in Porto Alegre from 2018 to 2025. Data were obtained from InfoDengue Brasil.
(PNG)

S6 Fig Spatial characterization of autochthonous dengue cases and DENV positive traps. Autochthonous dengue cases in Porto Alegre in 2022 (a), 2023 (b), 2024 (c) and 2025 (d). Autochthonous cases were assessed in the same districts in which positive trap analyses were performed. Proportion of dengue positive traps among total number of traps in 2022 (e), 2023 (f), 2024 (g) and 2025 (h). Base layer: https://prefeitura.poa.br/sites/default/files/usu_img/planejamento_urbano/Mapas%20Digitais/Shapefile_Bairros.zip
(PNG)

S7 Fig Weekly incidence of Zika virus (ZIKV) and chikungunya virus (CHIKV) in Porto Alegre, Brazil, from 2018 to 2025. Weekly incidence values of zika virus (ZIKV), yellow line (a), and chikungunya virus (CHIKV), blue line, in Porto Alegre from 2018 to 2025. Data were obtained from InfoDengue Brasil.
(PNG)

S8 Fig Observed and predicted weekly values obtained from rolling validation of the LASSO models and 95% prediction intervals. (a) Log-transformed dengue incidence rate per 100,000 inhabitants predicted using lagged entomological indicators, including the MFAI of *Aedes aegypti* and *Aedes albopictus*, Index P, and the VFII of both species, considering lags from 0 to 4 weeks. (b) Observed and predicted values of the MFAI of *Aedes aegypti* based on weekly climatic predictors and their respective lags (0–4 weeks), including cumulative weekly precipitation, mean temperature, and relative humidity. (c) Observed and predicted values of the MFAI of *Aedes albopictus* based on the same lagged climatic predictors (0–4 weeks). Black lines represent observed values, blue lines represent out-of-sample predictions generated during rolling validation, and gray shaded areas indicate empirical 95% prediction intervals derived from the distribution of residuals.
(PNG)

S1 Table Moran’s I of mosquito infestation per year. *** p < 0.001.
(DOCX)

S2 Table Kendall correlation of climate variables and mosquito infestation per district (2018–2025). * p < 0.05, ** p < 0.01.
(DOCX)

S3 Table Moran’s I of dengue cases per year. ** p < 0.01.
(DOCX)

S4 Table Moran’s I of DENV+ traps per year. ** p < 0.01, *** p < 0.001.
(DOCX)

S5 Table Correlation of *Aedes* proportion and autochthonous cases per year. ** p < 0.01, *** p < 0.001.
(DOCX)

S6 Table LASSO coefficients for MFAI *Aedes* model and dengue incidence model. (DOCX)

## References

[1] Guo C, Zhou Z, Wen Z, Liu Y, Zeng C, Xiao D, et al. Global epidemiology of dengue outbreaks in 1990-2015: a systematic review and meta-analysis. Front Cell Infect Microbiol. 2017;7:317. doi: 10.3389/fcimb.2017.00317 28748176 PMC5506197

[2] Bhatt S, Gething PW, Brady OJ, Messina JP, Farlow AW, Moyes CL, et al. The global distribution and burden of dengue. Nature. 2013;496(7446):504–7. doi: 10.1038/nature12060 23563266 PMC3651993

[3] Colón-González FJ, Sewe MO, Tompkins AM, Sjödin H, Casallas A, Rocklöv J, et al. Projecting the risk of mosquito-borne diseases in a warmer and more populated world: a multi-model, multi-scenario intercomparison modelling study. Lancet Planet Health. 2021;5(7):e404–14. doi: 10.1016/S2542-5196(21)00132-7 34245711 PMC8280459

[4] López MS, Jordan DI, Blatter E, Walker E, Gómez AA, Müller GV. Dengue emergence in the temperate Argentinian province of Santa Fe, 2009–2020. Sci Data. 2021;8(1):134. doi: 10.1038/s41597-021-00914-x 34016998 PMC8137689

[5] Shapiro LLM, Whitehead SA, Thomas MB. Quantifying the effects of temperature on mosquito and parasite traits that determine the transmission potential of human malaria. PLoS Biol. 2017;15(10):e2003489. doi: 10.1371/journal.pbio.2003489 29036170 PMC5658182

[6] Shocket MS, Verwillow AB, Numazu MG, Slamani H, Cohen JM, El Moustaid F. Transmission of West Nile and five other temperate mosquito-borne viruses peaks at temperatures between 23°C and 26°C. eLife. 2020. doi: 10.7554/eLife.58511 PMC7492091 32930091

[7] Delatte H, Gimonneau G, Triboire A, Fontenille D. Influence of temperature on immature development, survival, longevity, fecundity, and gonotrophic cycles of Aedes albopictus, vector of chikungunya and dengue in the Indian Ocean. J Med Entomol. 2009;46(1):33–41. doi: 10.1603/033.046.0105 19198515

[8] Samuel GH, Adelman ZN, Myles KM. Temperature-dependent effects on the replication and transmission of arthropod-borne viruses in their insect hosts. Curr Opin Insect Sci. 2016;16:108–13. doi: 10.1016/j.cois.2016.06.005 27720044 PMC5367266

[9] Neves DP. Parasitologia humana. 13 ed. Belo Horizonte: Atheneu; 2016.

[10] Lambrechts L, Paaijmans KP, Fansiri T, Carrington LB, Kramer LD, Thomas MB, et al. Impact of daily temperature fluctuations on dengue virus transmission by *Aedes aegypti*. Proc Natl Acad Sci U S A. 2011;108(18):7460–5. doi: 10.1073/pnas.1101377108 21502510 PMC3088608

[11] Carrington LB, Armijos MV, Lambrechts L, Scott TW. Fluctuations at a low mean temperature accelerate dengue virus transmission by *Aedes aegypti*. PLoS Negl Trop Dis. 2013;7(4):e2190. doi: 10.1371/journal.pntd.0002190 PMC3636080 23638208

[12] Iwamura T, Guzman-Holst A, Murray KA. Accelerating invasion potential of disease vector *Aedes aegypti* under climate change. Nat Commun. 2020;11(1):2130. doi: 10.1038/s41467-020-16010-4 32358588 PMC7195482

[13] Lwande OW, Obanda V, Lindström A, Ahlm C, Evander M, Näslund J. Globe-trotting *Aedes aegypti* and *Aedes albopictus*: risk factors for *Arbovirus Pandemics*. Vector-Borne and Zoonotic Diseases. 2020;20(2):71–81. doi: 10.1089/vbz.2019.2486 31556813 PMC7041325

[14] Franklinos LHV, Jones KE, Redding DW, Abubakar I. The effect of global change on mosquito-borne disease. Lancet Infect Dis. 2019;19(9):e302–12. doi: 10.1016/S1473-3099(19)30161-6 31227327

[15] Johnston CJ, Edwards AC, Vaux AGC, Abbott AJ, Hardy H, Wilson R, et al. Invasive mosquito surveillance in the United Kingdom 2020 to 2024: first detection of Aedes aegypti eggs in the UK and further detection of *Aedes albopictus*. PLOS Glob Public Health. 2025;5(10):e0004968. doi: 10.1371/journal.pgph.0004968 41032507 PMC12488007

[16] Kraemer MUG, Reiner RC Jr, Brady OJ, Messina JP, Gilbert M, Pigott DM, et al. Past and future spread of the arbovirus vectors *Aedes aegypti* and *Aedes albopictus*. Nat Microbiol. 2019;4(5):854–63. doi: 10.1038/s41564-019-0376-y 30833735 PMC6522366

[17] Forattini OP. Identificação de Aedes (Stegomyia) albopictus (Skuse) no Brasil. Rev Saude Publica. 1986;20(3):244–5. doi: 10.1590/S0034-89101986000300009 3809982

[18] Consoli RAGB, Oliveira RL de. Principais mosquitos de importância sanitária no Brasil. Editora FIOCRUZ; 1994. doi: 10.7476/9788575412909

[19] PAHO. Accessed 2026 March 14. https://www.paho.org/en/arbo-portal/dengue-data-and-analysis

[20] Brasil. Atualização de casos de arboviroses. 2026. Accessed 2026 March 17. https://www.gov.br/saude/pt-br/assuntos/saude-de-a-a-z/a/aedes-aegypti/monitoramento-das-arboviroses

[21] Lorenz C, Lemey P, Dellicour S. Dengue serotypes and epidemic dynamics in Brazil: a spatiotemporal perspective. Travel Med Infect Dis. 2026;69:102948. doi: 10.1016/j.tmaid.2025.102948 41418842

[22] Villabona-Arenas CJ, de Oliveira JL, Capra C de S, Balarini K, Loureiro M, Fonseca CRTP. Detection of four dengue serotypes suggests rise in hyperendemicity in urban centers of Brazil. PLoS Negl Trop Dis. 2014;8(2):e2620. doi: 10.1371/journal.pntd.0002620 PMC3937275 24587454

[23] Barcellos C, Matos V, Lana RM, Lowe R. Climate change, thermal anomalies, and the recent progression of dengue in Brazil. Sci Rep. 2024;14(1):5948. doi: 10.1038/s41598-024-56044-y 38467690 PMC10928122

[24] Lee SA, Economou T, de Castro Catão R, Barcellos C, Lowe R. The impact of climate suitability, urbanisation, and connectivity on the expansion of dengue in 21st century Brazil. PLoS Negl Trop Dis. 2021;15(12):e0009773. doi: 10.1371/journal.pntd.0009773 PMC8691609 34882679

[25] Codeco CT, Oliveira SS, Ferreira DAC, Riback TIS, Bastos LS, Lana RM, et al. Fast expansion of dengue in Brazil. Lancet Reg Health Am. 2022;12:100274. doi: 10.1016/j.lana.2022.100274 36776428 PMC9904033

[26] Copernicus Climate Change Service. ERA5-Land hourly data from 1950 to present. 2023. Accessed 2026 March 17. https://cds.climate.copernicus.eu

[27] Obolski U, Perez PN, Villabona-Arenas CJ, Thézé J, Faria NR, Lourenço J. MVSE: an R-package that estimates a climate-driven mosquito-borne viral suitability index. Methods Ecol Evol. 2019;10(8):1357–70. doi: 10.1111/2041-210X.13205 32391139 PMC7202302

[28] Souza CM, Shimbo Z, Rosa MR, Parente LL, Alencar A, Rudorff BFT. Reconstructing three decades of land use and land cover changes in Brazilian biomes with Landsat archive and Earth Engine. Remote Sens. 2020;12(17):2735. doi: 10.3390/rs12172735

[29] Codeco C, Coelho F, Cruz O, Oliveira S, Castro T, Bastos L. Infodengue: a nowcasting system for the surveillance of arboviruses in Brazil. Revue d’Épidémiologie et de Santé Publique. 2018;66:S386. doi: 10.1016/j.respe.2018.05.408

[30] Prefeitura de Porto Alegre. Onde está o Aedes? Painel epidemiológico. Porto Alegre: Secretaria Municipal de Saúde; 2025. https://prefeitura.poa.br/sms/onde-esta-o-aedes/dados-de-porto-alegre

[31] Moran PAP. Notes on continuous stochastic phenomena. Biometrika. 1950;37(1/2):17. doi: 10.2307/2332142 15420245

[32] da Cruz Ferreira DA, Freitas LP, Lowe R, Souza GD, Fujiwara RT, Martins Lana R. Introduction, establishment, and distribution of *Aedes aegypti* and dengue in a temperate capital of Brazil: a retrospective surveillance-based study. The Lancet Regional Health - Americas. 2025;48:101153. doi: 10.1016/j.lana.2025.101153 40620879 PMC12226368

[33] Chan YC, Chan KL, Ho BC. *Aedes aegypti* (L.) and *Aedes albopictus* (Skuse) in singapore city. 1. Distribution and density. Bull World Health Organ. 1971;44(5):617–27. 5316745 PMC2427851

[34] Braks MAH, Honório NA, Lourenço-De-Oliveira R, Juliano SA, Lounibos LP. Convergent habitat segregation of *Aedes aegypti* and *Aedes albopictus* (Diptera: Culicidae) in southeastern Brazil and Florida. J Med Entomol. 2003;40(6):785–94. doi: 10.1603/0022-2585-40.6.785 14765654

[35] Instituto Brasileiro de Geografia e Estatística IBGE. Censo demográfico 2022: resultados preliminares. Rio de Janeiro: IBGE; 2023. https://censo2022.ibge.gov.br/

[36] Mordecai EA, Cohen JM, Evans MV, Gudapati P, Johnson LR, Lippi CA. Detecting the impact of temperature on transmission of Zika, dengue, and chikungunya using mechanistic models. PLoS Negl Trop Dis. 2017;11(4):e0005568. doi: 10.1371/journal.pntd.0005568 PMC5423694 28448507

[37] Kramer IM, Kreß A, Klingelhöfer D, Scherer C, Phuyal P, Kuch U. Does winter cold really limit the dengue vector *Aedes aegypti* in Europe? Parasites & Vectors. 2020;13(1):178. doi: 10.1186/s13071-020-04054-w 32264941 PMC7140351

[38] Medley KA, Westby KM, Jenkins DG. Rapid local adaptation to northern winters in the invasive Asian tiger mosquito *Aedes albopictus*: a moving target. J Appl Ecol. 2019;56(11):2518–27. doi: 10.1111/1365-2664.13480

[39] Nakase T, Giovanetti M, Obolski U, Lourenço J. Global transmission suitability maps for dengue virus transmitted by *Aedes aegypti* from 1981 to 2019. Sci Data. 2023;10(1):275. doi: 10.1038/s41597-023-02170-7 37173303 PMC10182074

[40] Lee D-S, Park Y-S. Effects of cumulative temperature and precipitation patterns on mosquito abundance in urban Seoul, South Korea. Sci Rep. 2025;15(1):32790. doi: 10.1038/s41598-025-17975-2 40999045 PMC12464312

[41] Rey JR, O’Meara GF, O’Connell SM, Cutwa-Francis MM. Factors affecting mosquito production from stormwater drains and catch basins in two Florida cities. J Vector Ecol. 2006;31(2):334–43. doi: 10.3376/1081-1710(2006)31[334:fampfs]2.0.co;2 17249351

[42] Valdez LD, Sibona GJ, Diaz LA, Contigiani MS, Condat CA. Effects of rainfall on Culex mosquito population dynamics. J Theor Biol. 2017;421:28–38. doi: 10.1016/j.jtbi.2017.03.024 28351704

[43] Ng TC, Teo CH, Toh JY, Dunn AG, Ng CJ, Ang TF, et al. Factors influencing healthcare seeking in patients with dengue: systematic review. Trop Med Int Health. 2022;27(1):13–27. doi: 10.1111/tmi.13695 34655508

[44] Gregianini TS, Ranieri T, Favreto C, Nunes ZMA, Tumioto Giannini GL, Sanberg ND. Emerging arboviruses in Rio Grande do Sul, Brazil: Chikungunya and Zika outbreaks, 2014‐2016. Rev Med Virol. 2017;27(6). doi: 10.1002/rmv.1943 28929534

[45] Brasil. Atualização de casos de arboviroses. 2026. Accessed 2026 March 17. https://www.gov.br/saude/pt-br/assuntos/saude-de-a-a-z/a/aedes-aegypti/monitoramento-das-arboviroses

[46] Newman EA, Feng X, Onland JD, Walker KR, Young S, Smith K, et al. Defining the roles of local precipitation and anthropogenic water sources in driving the abundance of *Aedes aegypti*, an emerging disease vector in urban, arid landscapes. Sci Rep. 2024;14(1):2058. doi: 10.1038/s41598-023-50346-3 38267474 PMC10808563

