# Supplementary material for: Climate-driven spatiotemporal dynamics of Aedes infestation and dengue transmission in Porto Alegre, Southern Brazil
Source: PLoS Negl Trop Dis. 2026 Aug 24;20(8):e0014201. doi: 10.1371/journal.pntd.0014201 (PMC13537681; doi:10.1371/journal.pntd.0014201)
Supplement: Supplementary file 1 [file pntd.0014201.s009.docx]

| **Year** | ***Aedes aegypti*** | | ***Aedes albopictus*** | |
| --- | --- | --- | --- | --- |
|  | **Moran’s I** | **Z-score** | **Moran’s I** | **Z-score** |
| 2018 | 0.3717*** | 6.6302 | 0.3717*** | 4.9985 |
| 2019 | 0.5406*** | 9.4742 | 0.2859*** | 5.4709 |
| 2020 | 0.5518*** | 9.6615 | 0.3731*** | 7.1281 |
| 2021 | 0.4699*** | 8.2891 | 0.4220*** | 8.2377 |
| 2022 | 0.4900*** | 8.6716 | 0.2756*** | 5.3482 |
| 2023 | 0.6235*** | 10.91 | 0.4377*** | 9.5235 |
| 2024 | 0.5585*** | 9.796 | 0.3990*** | 8.4597 |
| 2025 | 0.5930*** | 10.399 | 0.3210*** | 6.0158 |

**Table S1**. Moran's I of mosquito infestation per year. *** p < 0.001.
