# Supplementary material for: Climate-driven spatiotemporal dynamics of Aedes infestation and dengue transmission in Porto Alegre, Southern Brazil
Source: PLoS Negl Trop Dis. 2026 Aug 24;20(8):e0014201. doi: 10.1371/journal.pntd.0014201 (PMC13537681; doi:10.1371/journal.pntd.0014201)
Supplement: Supplementary file 2 [file pntd.0014201.s010.docx]

| **District** | **Variables** | **τ** |
| --- | --- | --- |
| Boa Vista | Accumulated precipitation - *Aedes aegypti* | 0.6428* |
| Jardim Itu | Accumulated precipitation - *Aedes aegypti* | 0.7142* |
| Jardim Leopoldina | Accumulated precipitation - *Aedes aegypti* | 0.7857* |
| Jardim Sabará | Accumulated precipitation - *Aedes aegypti* | 0.6428* |
| Vila Jardim | Accumulated precipitation - *Aedes aegypti* | 0.7142** |
| Três Figueiras | Accumulated precipitation - *Aedes aegypti* | 0.7142** |
|  | Relative humidity - *Aedes aegypti* | 0.5714** |
| Mário Quintana | Accumulated precipitation - *Aedes aegypti* | 0.7857* |
| Partenon | Relative humidity - *Aedes albopictus* | 0.6428* |
| Sarandí | Accumulated precipitation - *Aedes albopictus* | 0.6428* |
| Teresópolis | Mean temperature - *Aedes albopictus* | 0.7142* |
|  | Relative humidity - *Aedes albopictus* | 0.6428* |
| Glória | Mean temperature - *Aedes albopictus* | 0.5714* |
|  | Relative humidity - *Aedes albopictus* | 0.6428* |

**Table S2.** Kendall correlation of climate variables and mosquito infestation per district (2018 - 2025). * p < 0.05, ** p < 0.01.
