# Supplementary material for: Climate-driven spatiotemporal dynamics of Aedes infestation and dengue transmission in Porto Alegre, Southern Brazil
Source: PLoS Negl Trop Dis. 2026 Aug 24;20(8):e0014201. doi: 10.1371/journal.pntd.0014201 (PMC13537681; doi:10.1371/journal.pntd.0014201)
Supplement: Supplementary file 3 [file pntd.0014201.s011.docx]

| **Year** | **Autochthonus cases** | |
| --- | --- | --- |
|  | **Moran’s I** | **Z-score** |
| 2025 | 0.1861** | 3.4954 |

**Table S3**. Moran's I of dengue cases per year. ** p < 0.01.
