# Supplementary material for: Climate-driven spatiotemporal dynamics of Aedes infestation and dengue transmission in Porto Alegre, Southern Brazil
Source: PLoS Negl Trop Dis. 2026 Aug 24;20(8):e0014201. doi: 10.1371/journal.pntd.0014201 (PMC13537681; doi:10.1371/journal.pntd.0014201)
Supplement: Supplementary file 4 [file pntd.0014201.s012.docx]

| **Year** | **DENV+ traps** | |
| --- | --- | --- |
|  | **Moran’s I** | **Z-score** |
| 2022 | 0.1384*** | 3.2264 |
| 2023 | 0.3085*** | 7.4082 |
| 2024 | 0.1445** | 2.9429 |
| 2025 | 0.5521*** | 10.044 |

**Table S4.** Moran's I of DENV+ traps per year. ** p < 0.01, *** p < 0.001.
