# Supplementary material for: Climate-driven spatiotemporal dynamics of Aedes infestation and dengue transmission in Porto Alegre, Southern Brazil
Source: PLoS Negl Trop Dis. 2026 Aug 24;20(8):e0014201. doi: 10.1371/journal.pntd.0014201 (PMC13537681; doi:10.1371/journal.pntd.0014201)
Supplement: Supplementary file 5 [file pntd.0014201.s013.docx]

| **Year** | **Variable** | **τ** |
| --- | --- | --- |
| 2022 | MFAI *- Aedes aegypti* - Autochthonous cases | 0.303* |
| 2024 | MFAI *- Aedes aegypti* - Autochthonous cases | 0.2942* |

**Table S5.** Correlation of Aedes proportion and autochthonus cases per year. ** p < 0.01, *** p < 0.001.
