# Supplementary material for: Climate-driven spatiotemporal dynamics of Aedes infestation and dengue transmission in Porto Alegre, Southern Brazil
Source: PLoS Negl Trop Dis. 2026 Aug 24;20(8):e0014201. doi: 10.1371/journal.pntd.0014201 (PMC13537681; doi:10.1371/journal.pntd.0014201)
Supplement: Supplementary file 6 [file pntd.0014201.s014.docx]

| **LASSO** | | | | | |
| --- | --- | --- | --- | --- | --- |
| **MFAI - *Aedes aegypti*** | | **MFAI - *Aedes albopictus*** | | **Dengue incidence per 100,000 inhabitants** | |
| **Variable** | **Coefficient** | **Variable** | **Coefficient** | **Variable** | **Coefficient** |
| (Intercept) | -3.4283 | (Intercept) | -0.0859 | (Intercept) | 0.6329 |
| Precipitation (mm) | -0.001 | Precipitation (mm) | -0,00003 | MFAI – *Aedes aegypti* | 0.5289 |
| Temperature (°C) | -0.0037 | Temperature (°C) | 0 | VFII - *Aedes aegypti* | -0.0372 |
| Humidity (%) | 0.0082 | Humidity (%) | 0.00016 | MFAI - *Aedes aegypti* (lag 1) | 0.654 |
| Precipitation (lag 1) | -0.0035 | Precipitation (lag 1) | -0.00006 | MFAI - *Aedes aegypti* (lag 2) | 0.7783 |
| Precipitation (lag 2) | -0.0022 | Precipitation (lag 2) | -0.00008 | MFAI - *Aedes aegypti* (lag 3) | 0.6804 |
| Precipitation (lag 3) | -0.003 | Precipitation (lag 3) | 0 | MFAI - Aedes aegypti (lag 4) | 1.2669 |
| Precipitation (lag 4) | -0.0008 | Precipitation (lag 4) | 0 | VFII - *Aedes aegypti* (lag 1) | -0.0144 |
| Temperature (lag 1) | 0.01327 | Temperature (lag 1) | 0.0002 | VFII - Aedes aegypti (lag 2) | -0.0306 |
| Temperature (lag 2) | 0.0101 | Temperature (lag 2) | 0.0004 | VFII - *Aedes aegypti* (lag 3) | 0 |
| Temperature (lag 3) | 0.0226 | Temperature (lag 3) | 0.0006 | VFII - *Aedes aegypti* (lag 4) | 0 |
| Temperature (lag 4) | 0.0419 | Temperature (lag 4) | 0.0009 | MFAI – *Aedes albopictus* | -6.738 |
| Humidity (lag 1) | 0.009 | Humidity (lag 1) | 0.0002 | VFII - *Aedes albopictus* | 1.3925 |
| Humidity (lag 2) | 0.0079 | Humidity (lag 2) | 0.0002 | MFAI - *Aedes albopictus* (lag 1) | -6.2065 |
| Humidity (lag 3) | 0.0092 | Humidity (lag 3) | 0.00007 | MFAI - *Aedes albopictus* (lag 2) | -7.1412 |
| Humidity (lag 4) | -0.0047 | Humidity (lag 4) | 0 | MFAI - *Aedes albopictus* (lag 3) | -8.616 |
|  |  |  |  | MFAI - *Aedes albopictus* (lag 4) | -7.1621 |
|  |  |  |  | VFII - *Aedes albopictus* (lag 1) | 0.8416 |
|  |  |  |  | VFII - *Aedes albopictus* (lag 2) | 0.6519 |
|  |  |  |  | VFII - *Aedes albopictus* (lag 3) | 0.1881 |
|  |  |  |  | VFII - *Aedes albopictus* (lag 4) | 0.4513 |
|  |  |  |  | IndexP | -0.0296 |
|  |  |  |  | IndexP (lag 1) | -0.0174 |
|  |  |  |  | IndexP (lag 2) | -0.0206 |
|  |  |  |  | IndexP (lag 3) | -0.0316 |
|  |  |  |  | IndexP (lag 4) | -0.0433 |

**Table S6**. LASSO coefficients for MFAI *Aedes* model and dengue incidence model**.**
