# Supplementary material for: Climate-driven spatiotemporal dynamics of Aedes infestation and dengue transmission in Porto Alegre, Southern Brazil
Source: PLoS Negl Trop Dis. 2026 Aug 24;20(8):e0014201. doi: 10.1371/journal.pntd.0014201 (PMC13537681; doi:10.1371/journal.pntd.0014201)
Supplement: Supplementary file 7 [file pntd.0014201.s016.docx]

PNTD-D-26-00625
Climate-driven spatiotemporal dynamics of Aedes infestation and dengue transmission in Porto Alegre, Southern Brazil.

We thank the reviewers for taking time to review our work, for their overall positive remarks and dedication to propose changes to the text, presentation and methodology. We have worked on applying changes to the manuscript in accordance with each of the reviewers’ comments and proposals. This has resulted in a more complete and clearer text, better figures and more adequate methodological implementation and presentation. Below we provide point-by-point responses to each comment. We refer the reviewers and the editor to the tracked version of the manuscript, which highlights in yellow all the changes to the original manuscript, including significant additions to text. To ease a second round of review, we include line numbers in the responses presented in this letter, but note that these numbers refer to the tracked version of the manuscript.

We hope that the revised manuscript is still considered of quality, value and interest to PLoS Neglected Diseases.

**Reviewers' Comments**

**Methods**

**Comment #1** - Reviewer #1: The study is methodologically sound, addresses a relevant and timely question, and uses an impressive, high-resolution dataset (2018–2025) from a under-studied temperate region.

**Comment #2** - Reviewer #2: To the best of my knowledge, the methods are appropriate for addressing the proposed research questions.

**Response**

We thank the reviewers for the positive comments above and constructive comments provided below. We have addressed the latter point-by-point, which has substantially increased the quality of results and their presentation.

**Comment #3** - Reviewer #3: The study design is generally appropriate for exploring the associations among climate, Aedes infestation, and dengue incidence. However, several methodological issues should be addressed. The methods are promising, but stronger justification, validation, and adjustment for key population factors are needed to support the conclusions.

First, the proposed Vector-Fitness Infestation Index (VFII = MFAI × Index P) needs stronger justification and validation. The authors should explain why a multiplicative index was used and whether the two components were standardized. They should also compare models using MFAI alone, Index P alone, and VFII to demonstrate whether VFII provides additional predictive value.

Second, the description of the LASSO models might be over optimistic. Additional performance metrics, such as MAE, R², calibration measures, and prediction intervals, should be reported. Because the data are time-series data, random ten-fold cross-validation may cause temporal information leakage. A time-based validation strategy, such as train-test split by year or rolling validation, might be more appropriate.

Third, the dengue models do not account for population size or population distribution. Since dengue burden is strongly affected by the exposed population, the authors should clarify whether case counts or incidence rates were modeled and whether population size was included as an offset, covariate, or standardization factor.

**Response**

We have considered the reviewer’s comments and have addressed them.

Regarding VFII, we now include explicit arguments, including the formulation and use of MFAI and index P (line 348) and an entirely new paragraph describing exercises and results that demonstrate VFII to be statistically more robust and informative than MFAI and index P alone (line 597). In summary, MFAI and index P were used in their original numerical scale when formulating VFII, and that MFAI was the most informative contributor to modelling.

Regarding the implementation of LASSO models, we agree with the reviewer on the need to provide additional performance outputs, and extra training and validation steps. As such, we have implemented significant changes to the LASSO models’ implementation and variety of outputs reported. These changes can be found throughout the text. Namely: we add and describe a rolling validation framework in model training (line 238); we include performance metrics (e.g. the paragraph starting in line 467, legend of Figure 5); we add a new Figure S8; and we add two discussion paragraphs (starting on line 559). Finally, we confirm that reported dengue cases were used as incidence (i.e. cases per 100K of population), thus considering the local landscape of population variation. We also confirm that we did not use local population size or distribution directly as modelled variables. We agree that it is relevant for these two points to be clear in the manuscript, and have added a paragraph in the discussion section dedicated to this (starting line 613).

**Results**

**Comment #4** - Reviewer #1: The results are scientifically sound and support the conclusions, but figure quality and a few missing analytical details reduce clarity.

**Response**

We note that four out of five figures have been updated and that significant analytical details have been added throughout the text, as a response to specific comments and proposals offered by the reviewers. We remain open to applying further changes to the figures or add more analytical detail that the reviewer feels could increase the quality of the visual and quantitative presentations.

**Comment #5** - Reviewer #2: The results are meaningful and well presented throughout the manuscript.

I have one comment that may induce an extension of the analysis: One of the key results was the stronger association between variables with higher lag. This was observed both between climatic variables and mosquito abundance (Table 1 all variables except for Ae. aegypti vs precipitation) and between mosquito abundance and dengue incidence (Table 2, all variables). Since the analysis was performed for lags between 0 and 4 weeks, I was left wondering if maximum correlation is reached at 4 weeks of lag, of if it keeps increasing at higher lags - which would be somewhat striking as a result. Would it be possible to extend the analysis for a few extra weeks of lag (even as a Supplementary analysis/table)? That could help readers with the same question I had when reading. If not feasible, please describe the reasons so readers understand the limitation of up to 4 weeks.

**Response**

We agree with the reviewer on the potential to increase the analyses beyond the four weeks. The approach and results have now been changed to include consideration of lag up to eight weeks (line 379 and Table 1; paragraph starting in line 437 and Table 2). In summary, results show that the highest gains in association between response and considered variables occurs in lags from zero to four weeks, after which gains in association are smaller (from four to eight weeks).

**Comment #6** - Reviewer #3: The results generally match the analysis plan described in the Methods. The authors present the entomological surveillance results, spatial clustering analyses, climate–mosquito associations, dengue incidence patterns, and predictive modeling results in a logical order. However, several minor issues should be addressed to improve clarity. First, the legend of Fig 2 is not sufficiently clear, and the figure legend should more explicitly explain what the mapped values represent. Second, Table S6 appears to use commas instead of decimal points in some numerical values. This should be corrected for consistency and readability.

**Response**

We agree with the reviewer and apologize for these small but relevant details that were missed in the original manuscript. We have now changed Figure 2 (visuals and legend), as well as the numerical values presented in Table S6.

**Conclusions**

**Comment #7** - Reviewer #1: The conclusions are largely supported by the data, and the public health relevance is clearly articulated.

**Response**

We thank the reviewer for the positive comment, and note that the conclusion section now includes a few additions and changes that increase its completeness and quality.

**Comment #8** - Reviewer #2: The discussed conclusions are supported by the presented results.
I only recommend authors to include a paragraph describing study limitations in the discussion, disclaiming any points that readers should be aware of before generalizing the result (e.g. single study site, data collection biases, etc.).

**Response**

We agree with the relevance of stating and describing more clearly the limitations of the current study. Given the format of the discussion section, we decide not to include a single limitations paragraph in the discussion section, but instead to explicitly state and discuss possible limitations throughout the discussion section (in paragraph starting in line 613)

**Comment #9** - Reviewer #3: The main conclusions are generally supported by the data presented. The manuscript clearly discusses how integrated climatic, entomological, and epidemiological surveillance data can improve our understanding of dengue risk in a temperate urban setting. The public health relevance is also well addressed, particularly regarding early warning, targeted vector control, and resource allocation. However, the conclusions related to Ae. albopictus should be stated more cautiously. The number of Ae. albopictus captured was much lower than that of Ae. aegypti, which may limit the robustness of correlation and modeling results for this species. In addition, the limitations of the analysis could be described more explicitly, particularly regarding model validation, possible reporting bias in dengue surveillance data, and the absence of population-related covariates.

**Response**

We agree that the manuscript would benefit from being more explicit about possible limitations and care for interpretation of presented results. As such, we have made a good number of changes to the discussion section. We have decided not to include a single limitations paragraph, but to include text regarding limitations across the discussion section, where and when appropriate. We now explicitly refer to limitations regarding, e.g.: observed versus predicted incidence ranges (paragraph starting in line 559); less certainty in ae. albopictus outputs (paragraph starting in line 571); not demonstrating validation exercises outside Porto Alegre (paragraph starting in line 613), and not including population variables directly in modelling (paragraph starting in line 613).

**Summary and General Comments**

**Comment #10 -** Reviewer #1: This manuscript by Silva and colleagues investigates the spatiotemporal relationships between climatic variables, Aedes mosquito infestation, and dengue incidence in Porto Alegre, Brazil, from 2018 to 2025. The study integrates high-resolution entomological surveillance data with climatic data and dengue case reports, employing spatial autocorrelation, Kendall correlation, polynomial regression, and LASSO regression. Overall, the study is methodologically sound and addresses a timely and relevant question. The dataset is rich, the analytical approaches are largely appropriate, and the public health implications are clearly articulated. However, several issues need to be addressed. In particular, the clarity of the presentation and the quality of the figures require improvement, as summarized below:

(1). ”climatic surveil in Porto Alegre" in Line 58 --"climatic surveillance in Porto Alegre"; (2) Please check the usage of 'Gloria' and 'Glória' in this manuscript and ensure consistent spelling throughout’; (3) Please check the usages in this manuscript and ensure consistent spelling throughout, for example, mosquitoes and mosquitos; (4) Panel labels (a, b, c, d) are present in figure 1 but not clearly distinguished from figure content. Also, check other figures; (5) Panels A, B, C are misaligned with the x-axis labels (0-5, 0-10, etc.) in figure 4. Please fix the layout; (6) The Methods section describes the use of ANOVA, but no related results are reported in the Results section.

**Response**

We sincerely thank the reviewer for these precise editorial and presentation observations. We have addressed all 6 points exactly as proposed:

Items (1), (2), and (3): The typo was corrected to "climatic surveillance" (line 1010), "Glória" was standardized with its diacritical mark across the text, and "mosquitoes/mosquitos" spelling variants were fully harmonized.

Items (4) and (5): Figure 1 panel labels (a, b, c, d) were visually isolated from content, and the alignment layout of Figure 4's x-axes and confidence bands was fully corrected.

Item (6): To maintain mathematical continuity and justify our model selection framework, the specific ANOVA results (F-statistics and p-values) evaluating the polynomial choices have been integrated directly within the Methods text under the Polynomial regression subsection (lines 212–227), mathematically supporting our structural choices.

**Comment #11 -** Reviewer #2: The manuscript describes a spatiotemporal analysis of Aedes species abundance and dengue incidence along with climate and land cover data, with focus on the Brazilian municipality of Porto Alegre. The study addresses a current and relevant issue, given the health burden created by the spatial expansion of dengue in the Americas. The manuscript is overall well written, well structured, and fits the scope of PLOS Neglected Tropical Diseases.

**Response**

We thank the reviewer for the constructive comments and proposals. We hope the reviewer finds added value to the changes and additions presented in the revised manuscript.

**Comment #12 -** Reviewer #3: This manuscript uses entomological surveillance, climatic data, and dengue case data from Porto Alegre between 2018 and 2025 to investigate climate-driven Aedes population dynamics and their relationship with dengue transmission. The authors also developed LASSO-based predictive models. The topic falls within the scope of PLOS NTD and has clear public health relevance.

However, its main findings, including the positive association between temperature and mosquito abundance, the influence of rainfall on mosquito density, and the several-week lag between mosquito indices and dengue cases, are broadly consistent with existing literature. The authors should further clarify the novelty of the study and strengthen the model construction, causal interpretation, and evaluation of predictive performance.

**Response**

We thank the constructive comments and proposals by the reviewer. We note, in particular, that in this revised version of the manuscript we have made changes regarding associations with variables and added extra material related to time lags, and how results seemingly fit observations from other studies. We also more explicitly discuss the relevance of this study, especially regarding the current emergence of mosquito-borne viruses in the southern states of Brazil and the dearth of studies in the region of Porto Alegre. We believe that addressing reviewers’ comments and raised issues, resulting in substantial changes to figures and text, has increased the quality of the manuscript.
