# Supplementary material for: Climate-driven spatiotemporal dynamics of Aedes infestation and dengue transmission in Porto Alegre, Southern Brazil
Source: PLoS Negl Trop Dis. 2026 Aug 24;20(8):e0014201. doi: 10.1371/journal.pntd.0014201 (PMC13537681; doi:10.1371/journal.pntd.0014201)
Supplement: Peer review history

# Peer review: Decision Letter 0

**DOI:** 10.1371/journal.pntd.0014201.r001

**Authors:** Audrey Lenhart, Quanhui Liu

16 Jun 2026

PNTD-D-26-00625

Climate-driven spatiotemporal dynamics of Aedes infestation and dengue transmission in Porto Alegre, Southern Brazil.

PLOS Neglected Tropical Diseases

Dear Dr. Cupertino de Freitas,

Thank you for submitting your manuscript to PLOS Neglected Tropical Diseases. After careful consideration, we feel that it has merit but does not fully meet PLOS Neglected Tropical Diseases's publication criteria as it currently stands. Therefore, we invite you to submit a revised version of the manuscript that addresses the points raised during the review process.

Please submit your revised manuscript within by Aug 15 2026 11:59PM. If you will need more time than this to complete your revisions, please reply to this message or contact the journal office at plosntds@plos.org. When you're ready to submit your revision, log on to https://www.editorialmanager.com/pntd/ and select the 'Submissions Needing Revision' folder to locate your manuscript file.

Please include the following items when submitting your revised manuscript:

* A letter that responds to each point raised by the editor and reviewer(s). You should upload this letter as a separate file labeled 'Response to Reviewers'. This file does not need to include responses to any formatting updates and technical items listed in the 'Journal Requirements' section below.

* A marked-up copy of your manuscript that highlights changes made to the original version. You should upload this as a separate file labeled 'Revised Manuscript with Track Changes'.

* An unmarked version of your revised paper without tracked changes. You should upload this as a separate file labeled 'Manuscript'.

If you would like to make changes to your financial disclosure, competing interests statement, or data availability statement, please make these updates within the submission form at the time of resubmission. Guidelines for resubmitting your figure files are available below the reviewer comments at the end of this letter.

As the corresponding author, your ORCID iD is verified in the submission system and will appear in the published article. PLOS supports the use of ORCID, and we encourage all coauthors to register for an ORCID iD and use it as well. Please encourage your coauthors to verify their ORCID iD within the submission system before final acceptance, as unverified ORCID iDs will not appear in the published article. Only the individual author can complete the verification step; PLOS staff cannot verify ORCID iDs on behalf of authors.

We look forward to receiving your revised manuscript.

Kind regards,

Quanhui Liu, Ph.D.

Academic Editor

PLOS Neglected Tropical Diseases

Audrey Lenhart

Section Editor

PLOS Neglected Tropical Diseases

Shaden Kamhawi

co-Editor-in-Chief

PLOS Neglected Tropical Diseases

orcid.org/0000-0003-4304-636XX

Paul Brindley

co-Editor-in-Chief

PLOS Neglected Tropical Diseases

orcid.org/0000-0003-1765-0002

**Additional Editor Comments (if provided):**

**Journal Requirements:**

If the reviewer comments include a recommendation to cite specific previously published works, please review and evaluate these publications to determine whether they are relevant and should be cited. There is no requirement to cite these works unless the editor has indicated otherwise.

1) We ask that a manuscript source file is provided at Revision. Please upload your manuscript file as a .doc, .docx, .rtf or .tex. If you are providing a .tex file, please upload it under the item type u2018LaTeX Source Fileu2019 and leave your .pdf version as the item type u2018Manuscriptu2019.

2) Please upload all main figures as separate Figure files in .tif or .eps format. For more information about how to convert and format your figure files please see our guidelines:

https://journals.plos.org/plosntds/s/figures

3) Please upload a copy of Figure Fig 1-5 which you refer to in your text on pages 11, 12, 14, 17, and 21. Or, if the figure is no longer to be included as part of the submission please remove all reference to it within the text.

4) We notice that your supplementary Figures, and Tables are included in the manuscript file. Please remove them and upload them with the file type 'Supporting Information'. Please ensure that each Supporting Information file has a legend listed in the manuscript after the references list.

5) In the online submission form, you indicated that Entomological data from MosquiTRAP surveillance were obtained under institutional agreements and ethical approval from the Research Ethics Committee of the Municipal Health Department of Porto Alegre (CEP SMSPA). Due to these restrictions, raw data are not publicly available. Data can be made available from the corresponding author upon reasonable request and with permission from the relevant authorities.. All PLOS journals now require all data underlying the findings described in their manuscript to be freely available to other researchers, either

1. In a public repository

2. Within the manuscript itself

3. Uploaded as supplementary information.

This policy applies to all data except where public deposition would breach compliance with the protocol approved by your research ethics board. If your data cannot be made publicly available for ethical or legal reasons (e.g., public availability would compromise patient privacy), please explain your reasons by return email and your exemption request will be escalated to the editor for approval. Your exemption request will be handled independently and will not hold up the peer review process, but will need to be resolved should your manuscript be accepted for publication. One of the Editorial team will then be in touch if there are any issues.

**Reviewers' Comments:**

Reviewer's Responses to Questions

**Key Review Criteria Required for Acceptance?**

As you describe the new analyses required for acceptance, please consider the following:

**Methods**

-Are the objectives of the study clearly articulated with a clear testable hypothesis stated?

-Is the study design appropriate to address the stated objectives?

-Is the population clearly described and appropriate for the hypothesis being tested?

-Is the sample size sufficient to ensure adequate power to address the hypothesis being tested?

-Were correct statistical analysis used to support conclusions?

-Are there concerns about ethical or regulatory requirements being met?

Reviewer #1: The study is methodologically sound, addresses a relevant and timely question, and uses an impressive, high-resolution dataset (2018–2025) from a under-studied temperate region.

Reviewer #2: To the best of my knowledge, the methods are appropriate for addressing the proposed research questions.

Reviewer #3: The study design is generally appropriate for exploring the associations among climate, Aedes infestation, and dengue incidence. However, several methodological issues should be addressed. The methods are promising, but stronger justification, validation, and adjustment for key population factors are needed to support the conclusions.

First, the proposed Vector-Fitness Infestation Index (VFII = MFAI × Index P) needs stronger justification and validation. The authors should explain why a multiplicative index was used and whether the two components were standardized. They should also compare models using MFAI alone, Index P alone, and VFII to demonstrate whether VFII provides additional predictive value.

Second, the description of the LASSO models might be over optimistic. Additional performance metrics, such as MAE, R², calibration measures, and prediction intervals, should be reported. Because the data are time-series data, random ten-fold cross-validation may cause temporal information leakage. A time-based validation strategy, such as train-test split by year or rolling validation, might be more appropriate.

Third, the dengue models do not account for population size or population distribution. Since dengue burden is strongly affected by the exposed population, the authors should clarify whether case counts or incidence rates were modeled and whether population size was included as an offset, covariate, or standardization factor.

**********

**Results**

-Does the analysis presented match the analysis plan?

-Are the results clearly and completely presented?

-Are the figures (Tables, Images) of sufficient quality for clarity?

Reviewer #1: The results are scientifically sound and support the conclusions, but figure quality and a few missing analytical details reduce clarity.

Reviewer #2: The results are meaningful and well presented throughout the manuscript. I have one comment that may induce an extension of the analysis:

One of the key results was the stronger association between variables with higher lag. This was observed both between climatic variables and mosquito abundance (Table 1 all variables except for Ae. aegypti vs precipitation) and between mosquito abundance and dengue incidence (Table 2, all variables). Since the analysis was performed for lags between 0 and 4 weeks, I was left wondering if maximum correlation is reached at 4 weeks of lag, of if it keeps increasing at higher lags - which would be somewhat striking as a result.

Would it be possible to extend the analysis for a few extra weeks of lag (even as a Supplementary analysis/table)? That could help readers with the same question I had when reading. If not feasible, please describe the reasons so readers understand the limitation of up to 4 weeks.

Reviewer #3: The results generally match the analysis plan described in the Methods. The authors present the entomological surveillance results, spatial clustering analyses, climate–mosquito associations, dengue incidence patterns, and predictive modeling results in a logical order.

However, several minor issues should be addressed to improve clarity. First, the legend of Fig 2 is not sufficiently clear, and the figure legend should more explicitly explain what the mapped values represent. Second, Table S6 appears to use commas instead of decimal points in some numerical values. This should be corrected for consistency and readability.

**********

**Conclusions**

-Are the conclusions supported by the data presented?

-Are the limitations of analysis clearly described?

-Do the authors discuss how these data can be helpful to advance our understanding of the topic under study?

-Is public health relevance addressed?

Reviewer #1: The conclusions are largely supported by the data, and the public health relevance is clearly articulated.

Reviewer #2: The discussed conclusions are supported by the presented results.

I only recommend authors to include a paragraph describing study limitations in the discussion, disclaiming any points that readers should be aware of before generalizing the result (e.g. single study site, data collection biases, etc.).

Reviewer #3: The main conclusions are generally supported by the data presented. The manuscript clearly discusses how integrated climatic, entomological, and epidemiological surveillance data can improve our understanding of dengue risk in a temperate urban setting. The public health relevance is also well addressed, particularly regarding early warning, targeted vector control, and resource allocation.

However, the conclusions related to Ae. albopictus should be stated more cautiously. The number of Ae. albopictus captured was much lower than that of Ae. aegypti, which may limit the robustness of correlation and modeling results for this species.

In addition, the limitations of the analysis could be described more explicitly, particularly regarding model validation, possible reporting bias in dengue surveillance data, and the absence of population-related covariates.

**********

**Editorial and Data Presentation Modifications?**

Use this section for editorial suggestions as well as relatively minor modifications of existing data that would enhance clarity. If the only modifications needed are minor and/or editorial, you may wish to recommend “Minor Revision” or “Accept”.

Reviewer #1: Minor Revision

The manuscript is scientifically sound and well-organized. The required changes are primarily editorial (typos, diacritical marks, formatting) and data presentation improvements (figure alignment, table restructuring, supplementary table clarity).

Reviewer #2: I have no recommended editorial or data modifications.

Reviewer #3: Minor Revision

**********

**Summary and General Comments**

Use this section to provide overall comments, discuss strengths/weaknesses of the study, novelty, significance, general execution and scholarship. You may also include additional comments for the author, including concerns about dual publication, research ethics, or publication ethics. If requesting major revision, please articulate the new experiments that are needed.

Reviewer #1: This manuscript by Silva and colleagues investigates the spatiotemporal relationships between climatic variables, Aedes mosquito infestation, and dengue incidence in Porto Alegre, Brazil, from 2018 to 2025. The study integrates high-resolution entomological surveillance data with climatic data and dengue case reports, employing spatial autocorrelation, Kendall correlation, polynomial regression, and LASSO regression. Overall, the study is methodologically sound and addresses a timely and relevant question. The dataset is rich, the analytical approaches are largely appropriate, and the public health implications are clearly articulated. However, several issues need to be addressed. In particular, the clarity of the presentation and the quality of the figures require improvement, as summarized below:

1. ”climatic surveil in Porto Alegre" in Line 58 --"climatic surveillance in Porto Alegre"

2. Please check the usage of 'Gloria' and 'Glória' in this manuscript and ensure consistent spelling throughout.

3. Please check the usages in this manuscript and ensure consistent spelling throughout, for example, mosquitoes and mosquitos.

4. Panel labels (a, b, c, d) are present in figure 1 but not clearly distinguished from figure content. Also, check other figures.

5. Panels A, B, C are misaligned with the x-axis labels (0-5, 0-10, etc.) in figure 4. Please fix the layout.

6. The Methods section describes the use of ANOVA, but no related results are reported in the Results section.

Reviewer #2: The manuscript describes a spatiotemporal analysis of Aedes species abundance and dengue incidence along with climate and land cover data, with focus on the Brazilian municipality of Porto Alegre. The study addresses a current and relevant issue, given the health burden created by the spatial expansion of dengue in the Americas. The manuscript is overall well written, well structured, and fits the scope of PLOS Neglected Tropical Diseases.

Reviewer #3: This manuscript uses entomological surveillance, climatic data, and dengue case data from Porto Alegre between 2018 and 2025 to investigate climate-driven Aedes population dynamics and their relationship with dengue transmission. The authors also developed LASSO-based predictive models. The topic falls within the scope of PLOS NTD and has clear public health relevance.

However, its main findings, including the positive association between temperature and mosquito abundance, the influence of rainfall on mosquito density, and the several-week lag between mosquito indices and dengue cases, are broadly consistent with existing literature. The authors should further clarify the novelty of the study and strengthen the model construction, causal interpretation, and evaluation of predictive performance.

**********

PLOS authors have the option to publish the peer review history of their article (what does this mean?). If published, this will include your full peer review and any attached files.

If you choose “no”, your identity will remain anonymous but your review may still be made public.

**Do you want your identity to be public for this peer review?**  For information about this choice, including consent withdrawal, please see our Privacy Policy.

Reviewer #1: No

Reviewer #2: No

Reviewer #3: No

[NOTE: If reviewer comments were submitted as an attachment file, they will be attached to this email and accessible via the submission site. Please log into your account, locate the manuscript record, and check for the action link "View Attachments". If this link does not appear, there are no attachment files.]

**Figure resubmission:**

**Reproducibility:**

To enhance the reproducibility of your results, we recommend that authors of applicable studies deposit laboratory protocols in protocols.io, where a protocol can be assigned its own identifier (DOI) such that it can be cited independently in the future. Additionally, PLOS ONE offers an option to publish peer-reviewed clinical study protocols. Read more information on sharing protocols at https://plos.org/protocols?utm_medium=editorial-email&utm_source=authorletters&utm_campaign=protocols

# Author comment: Author response to Decision Letter 1

**DOI:** 10.1371/journal.pntd.0014201.r002

17 Jul 2026

Attachment Submitted filename: Response to Reviewers.docx

# Editor report: Decision Letter 1

**DOI:** 10.1371/journal.pntd.0014201.r003

**Authors:** Audrey Lenhart, Quanhui Liu

6 Aug 2026

Dear Ms Cupertino de Freitas,

We are pleased to inform you that your manuscript 'Climate-driven spatiotemporal dynamics of Aedes infestation and dengue transmission in Porto Alegre, Southern Brazil.' has been provisionally accepted for publication in PLOS Neglected Tropical Diseases.

Before your manuscript can be formally accepted you will need to complete some formatting changes, which you will receive in a follow up email. A member of our team will be in touch with a set of requests.

Please note that your manuscript will not be scheduled for publication until you have made the required changes, so a swift response is appreciated.

IMPORTANT: The editorial review process is now complete. PLOS will only permit corrections to spelling, formatting or significant scientific errors from this point onwards. Requests for major changes, or any which affect the scientific understanding of your work, will cause delays to the publication date of your manuscript.

Should you, your institution's press office or the journal office choose to press release your paper, you will automatically be opted out of early publication. We ask that you notify us now if you or your institution is planning to press release the article. All press must be co-ordinated with PLOS.

Thank you again for supporting Open Access publishing; we are looking forward to publishing your work in PLOS Neglected Tropical Diseases.

Best regards,

Audrey Lenhart

Section Editor

PLOS Neglected Tropical Diseases

Audrey Lenhart

Section Editor

PLOS Neglected Tropical Diseases

Shaden Kamhawi

co-Editor-in-Chief

PLOS Neglected Tropical Diseases

orcid.org/0000-0003-4304-636XX

Paul Brindley

co-Editor-in-Chief

PLOS Neglected Tropical Diseases

orcid.org/0000-0003-1765-0002

***********************************************************

# Editor report: Acceptance letter

**DOI:** 10.1371/journal.pntd.0014201.r004

**Authors:** Audrey Lenhart, Quanhui Liu

Dear Ms Cupertino de Freitas,

We are delighted to inform you that your manuscript, "Climate-driven spatiotemporal dynamics of Aedes infestation and dengue transmission in Porto Alegre, Southern Brazil.," has been formally accepted for publication in PLOS Neglected Tropical Diseases.

We have now passed your article onto the PLOS Production Department who will complete the rest of the publication process. All authors will receive a confirmation email upon publication.

The corresponding author will soon be receiving a typeset proof for review, to ensure errors have not been introduced during production. Please review the PDF proof of your manuscript carefully, as this is the last chance to correct any scientific or type-setting errors. Please note that major changes, or those which affect the scientific understanding of the work, will likely cause delays to the publication date of your manuscript. Note: Proofs for Front Matter articles (Editorial, Viewpoint, Symposium, Review, etc...) are generated on a different schedule and may not be made available as quickly.

Soon after your final files are uploaded, the early version of your manuscript will be published online unless you opted out of this process. The date of the early version will be your article's publication date. The final article will be published to the same URL, and all versions of the paper will be accessible to readers.

For Research Articles, you will receive an invoice from PLOS for your publication fee after your manuscript has reached the completed accept phase. If you receive an email requesting payment before acceptance or for any other service, this may be a phishing scheme. Learn how to identify phishing emails and protect your accounts at https://explore.plos.org/phishing.

Thank you again for supporting open-access publishing; we are looking forward to publishing your work in PLOS Neglected Tropical Diseases.

Best regards,

Shaden Kamhawi

co-Editor-in-Chief

PLOS Neglected Tropical Diseases

Paul Brindley

co-Editor-in-Chief

PLOS Neglected Tropical Diseases
